# Exploring the complex population structure and admixture of four local Hungarian sheep breeds

**DOI:** 10.3389/fgene.2025.1507315

**Published:** 2025-03-19

**Authors:** Johanna Ramírez-Díaz, Tania Bobbo, Bernt Guldbrandtsen, Anna A. Schönherz, Paolo Cozzi, Szilvia Kusza, Goutam Sahana, Alessandra Stella, Arianna Manunza

**Affiliations:** ^1^ Institute of Agricultural Biology and Biotechnology, National Research Council, Milan, Italy; ^2^ Institute for Biomedical Technologies, National Research Council (CNR), Segrate, Italy; ^3^ Department of Veterinary and Animal Sciences, University of Copenhagen, Copenhagen, Denmark; ^4^ Department of Animal and Veterinary Sciences, Aarhus University, Aarhus, Denmark; ^5^ Centre for Agricultural Genomics and Biotechnology, University of Debrecen, Debrecen, Hungary; ^6^ Center for Quantitative Genetics and Genomics, Aarhus University, Aarhus, Denmark

**Keywords:** admixture, demography, genetic structure, Hungarian sheep, local sheep breeds

## Abstract

The origin of sheep and their spread following domestication have been widely investigated using archaeology, genetics, and genomics. A thorough investigation of the genetic diversity of the breeds is key to providing useful information for conservation and breeding programmes. In Hungary, sheep farming contributes to the agricultural sector and national economy. It plays a crucial role in rural livelihoods, exports, and environmental management while also contributing to the national economy and preserving Hungary’s cultural and agricultural heritage through traditional breeds. This study aims to analyse the population structure and patterns of admixture in four local Hungarian sheep breeds, namely, Indigenous Tsigai, Hortobagyi Racka, Cikta, and Bábolna Tetra. Our results revealed that the indigenous Hungarian Hortobagyi Racka sheep are distinct from the other Hungarian breeds studied. The effective population sizes were found to be low, with varying levels of genomic inbreeding both within and across breeds. These results align with documented bottlenecks and instances of crossbreeding with other local or improved breeds. Ancestry analysis demonstrated some introgression between Scandinavian and Hungarian sheep breeds and *vice versa*. This gene flow may have occurred recently due to the widespread use of northern breeds such as Finnsheep and Romanov to enhance productivity, but it could also date back much further. Despite some limitations, our outcomes can contribute to future conservation plans, and a more comprehensive analysis of all native Hungarian sheep breeds should be highlighted to the relevant authorities in order to secure further funds.

## 1 Introduction

Elucidating the genetic composition and the origin of local breeds is essential to maintaining global genetic biodiversity via appropriate management plans, particularly in light of the modern environmental crisis and rapidly changing climatic conditions. Several studies have described the global genetic diversity of sheep using genotype data (Ceccobelli et al., 2023; Wanjala et al., 2023; Zsolnai et al., 2021; Ciani et al., 2020; Deniskova et al., 2018; Kijas et al., 2012; Zhao et al., 2017), but research on breed origin and history in Central–Eastern Europe is still in its early stages and is mostly based on microsatellites or mitochondrial data (Loukovitis et al., 2023; Astuti et al., 2023a; Gáspárdy et al., 2021; Dudu et al., 2016; Kawęcka et al., 2022; Kusza et al., 2008; 2009; 2010; 2011; Rannamäe et al., 2023). Hungary’s vast pasturelands and moderate climate provide a favourable environment for animal production. Hence, agriculture plays an important economic role, especially in rural areas (Bakucs et al., 2020). Agriculture employs 4.8% of Hungary’s workforce, and in livestock production, sheep rank third in economic significance after poultry and pigs (Astuti et al., 2023b; https://www.ksh.hu/?lang=en). In this study, we analysed genotype data from four local Hungarian sheep breeds, namely, indigenous Tsigai, Hortobagyi Racka, Cikta, and Bábolna Tetra. All these breeds are long-tailed. The Indigenous Tsigai is a traditional sheep breed that belongs to the Tsigai group of breeds, indigenous to Eastern and Central Europe (Astuti et al., 2023a; Kusza et al., 2011; 2008). The Hortobagyi Racka sheep is an ancient Hungarian breed known for its striking spiral-shaped horns, which are present in both male and female sheep. This breed is well-adapted to harsh environmental conditions, thriving in both hot summers and cold winters. The breed comes in two distinct colour variants, namely, black and white. They are believed to be similar to the Zackel sheep of ancient Egypt, which were the origin of various migrations to the Middle East and Europe (Draganescu and Grosu, 2010). Phenotypically, breeds from the Zackel group are small to medium-sized, with long, coarse wool and spiralled horns (Kusza et al., 2008). Cikta historically belongs to the “mountain sheep breeds,” a group considered to be relics of the extinct “Zaupel or Neolithic Turf sheep,” which, in turn, was one of the original breeds of the European Alps (Gáspárdy et al., 2021; Heinz et al., 2021; Kovács et al., 2019). Bábolna Tetra is a white, prolific breed developed from the Hungarian Merino, the Romanov, and the Finnish Landrace, combining a “Nordic” background and Hungarian influence (Neubauer et al., 2015). During the last two centuries, Merino and Texel have contributed genetically to several Hungarian breeds (Beynon et al., 2015; Ciani et al., 2020). Following Mujitaba et al. (2024), the local risk status of these Hungarian sheep breeds is as follows: the Hungarian Hortobagyi Racka (both varieties) and Indigenous Tsigai are categorised as vulnerable, while Cikta was categorised as endangered. It is, therefore, essential to conserve the genetic resources of these important local sheep. The decline in the populations of these breeds was predominantly driven by a shift in breeders’ preference towards more productive and modern breeds. However, several conservation programmes, both *in situ* and *ex situ*, have already been developed to support their preservation and promote their restoration (Mujitaba et al., 2024). The primary objective of this study was to evaluate the genomic diversity of four indigenous Hungarian sheep breeds in comparison to other Central–Eastern European breeds using genotyping data, with the goal of clarifying their origins and filling the knowledge gap of this poorly explored group of breeds. The research further included local breeds from neighbouring regions to examine gene flow and historical admixture events that occurred during the Viking Age and early Middle Ages (800–1300 CE), a period marked by extensive Norse influence across Europe, spanning from Iceland to Russia (Ellis, 2021). These findings provide valuable insights into the population structure and genetic connections that have shaped contemporary Hungarian sheep breeds.

## 2 Materials and methods

Due to the sampling framework of the SMARTER project, only the breeds included in the present analysis have been genotyped. However, in the FAO DAD-IS system (https://www.fao.org/dad-is/browse-by-country-and-species/en/), a total of 33 sheep breeds are listed as both local and commercial. Three Hungarian sheep breeds were newly genotyped for this study in the context of the SMARTER project (https://www.smarterproject.eu/): Hortobagyi Racka (28 animals from 2 farms; n = 16 white and n = 12 black), Indigenous Tsigai (34 animals from 2 farms), and Bábolna Tetra (27 animals from 1 farm). The total blood samples were collected from the jugular veins, preserved in tubes containing EDTA, and transferred to FTA cards. The dried FTA cards were kept at room temperature until they were shipped to the genotyping company for further processing. The entire workflow, including DNA isolation, genotyping, and the conversion of raw signals into Ovine 50K genotypes, was outsourced to Neogen Chemicals Limited (https://www.neogen.com). Neogen utilised the Infinium^®^ OvineSNP50 BeadChip Array, which contains over 50,000 evenly distributed SNP-targeting probes. Genotype data for the remaining breeds were obtained from publicly available databases (see Supplementary Table 1 for details). We kept the new Hortobagyi Racka data separate from the previously genotyped Racka (Barbato et al., 2017) in order to assess their genetic structure and highlight any potential differences between these two populations. Genotype data for Cikta were also obtained from a publicly available database (Ciani et al., 2020). Sampling locations are detailed in Supplementary Figure S1.

To represent Merino and Texel influences, we included data from those breeds in the F_ST_ and individual ancestry (genetic components) estimations to identify “introgression” and estimate a genetic contribution score (GCS, see below).

In total, genotype data for 1,338 sheep were available. All data were genotyped using the Illumina OvineSNP50 or the Illumina Ovine HD SNP (Supplementary Table S1). After merging data using PLINK v1.9 (Chang et al., 2015), the SNP data were mapped against the genome assembly Oar_v3.1 using SNPchiMp v.3 (Nicolazzi et al., 2015) and a series of custom scripts developed in the context of the SMARTER project (https://smarter-database.readthedocs.io/en/latest/index.html). Quality control was performed using PLINK v2.0 (Chang et al., 2015), following the FAO guidelines for the genomic characterisation of animal genetic resources (Ajmone-Marsan et al., 2023). Variants with a call rate below 90% and a minor allele frequency of less than 1% were excluded from the analysis. Individuals with genotype calls of less than 95% markers were also removed. Additionally, individuals displaying extreme levels of observed heterozygosity were filtered out using the “--het flag” for obtaining the genotypic counts. Afterwards, we used R v4.1 (R Core Team, 2018) to first compute the observed heterozygosity and then identify those individuals having within-population extreme heterozygosity (>3 SD). Linkage disequilibrium (LD) pruning was performed using PLINK v1.9 with a window size of 50 SNPs, shifting the windows by five SNPs at each step, and an r^2 threshold of 0.5 to reduce ascertainment bias (Lachance and Tishkoff, 2013). Duplicate samples and closely related individuals were removed by applying the command “--king cat-off” in PLINK v2.0 (Chang et al., 2015). Unmapped variants and variants on the sex chromosomes were also excluded. After quality control, a total of 31,010 SNPs were used for genetic diversity and population structure analyses. To avoid the loss of information due to the LD pruning process when applying the analysis based on LD, we reintroduced the variants in linkage disequilibrium to our filtered dataset, resulting in a total of 35,688 SNPs for the runs of homozygosity (ROH) detection and $N_{e}$ estimation. Finally, to reduce possible bias due to the unbalanced number of animals per population, we performed a selection of individuals based on population structure using BITEv1 (Milanesi et al., 2017), retaining at least five and at most 30 animals per breed, leaving a total of 763 individuals for following analyses. Since we used a suite of different programmes that require different input file formats, the appropriate conversions were performed using PGDSpider v2.0.4.0 (Lischer and Excoffier, 2012).

### 2.1 Population structure and demographic analyses

Population differentiation and genetic variability among and within breeds, as well as among groups of breeds, were assessed using Wright’s F_ST_ and Reynolds’ genetic distance (Supplementary Table S2; Supplementary Figure S2). For the visualisation of pairwise F_ST_ values, we used the R package pheatmap v. 1.0.12 (https://CRAN.R-project.org/package=pheatmap, R Core Team, 2018).

Software GONE (Santiago et al., 2020) was used to obtain contemporary and historical estimates of the effective population size with unphased genotypes, correcting for sample sizes, and using the default settings (except for the number of internal replicates, which was set to 100). The so-called contemporary $N_{e}$ is the average effective population size in the most recent generations (10 in our study). In addition, we provided genetic map information (Prieur et al., 2017) to improve the performance in estimating $N_{e}$ , as recommended by the authors (Santiago et al., 2020) and by a recent simulation study (Santiago et al., 2024). Time intervals in the number of generations were converted into years, assuming 3 years per generation (Miranda et al., 2023).

Runs of homozygosity were detected using the R package detectRUNS 0.9.5 (Biscarini et al., 2018) with default settings, except for the minimum length of homozygous segments and the minimum number of SNPs included in the runs (set to 1 Mb and 50 SNP, respectively). The genomic inbreeding coefficient (FROH) was computed as the total length of the genome covered by ROH divided by the total length of autosomes flanked by markers. Their distribution per classes of length (0–2, 2–4, 4–8, 8–16, and >16 Mb) was determined.

Clustering analysis was used to investigate population structure and genetic components at the individual level, applying the principal component analysis (PCA) using the SNPRelate package v. 1.38 (Zheng et al., 2012) and the unsupervised method in ADMIXTURE v.1.3 (Alexander et al., 2009), with K clusters ranging from 2 to 40. The cross-validation error function was computed for each value of K. K with the lowest cross-validation error was chosen to model the data. We used evalAdmix v. 0.95 (Garcia-Erill and Albrechtsen, 2020; available at https://github.com/GenisGE/evalAdmix) to evaluate the fit of admixture proportions. In brief, the method estimates the correlation of the residual difference between the true genotypes and the genotypes predicted by the model. In the case of poor fit of the admixture model, individuals with similar demographic histories exhibit a positive correlation with their residuals. The correlation of the residuals’ matrix was plotted using R v4.1 (R Core Team, 2018) and the visFuns.R script available with the program and BITE was used to plot the ADMIXTURE results. In addition to established traditional methods of population analysis, we applied the pipeline implemented in NetViewP (Neuditschko et al., 2012; Steinig et al., 2016) (https://github.com/esteinig/netviewr/tree/master/R) and used NetViewR as a visualisation tool. This programme offers a more comprehensive overview of the population structure at different levels of genetic similarity, allowing a focus on fine- or large-scale structure (high-resolution population structure). The programme NetViewP implements three machine learning methods to establish the mutual k-nearest neighbour threshold for network analysis. Since the dataset included several breeds with a critical conservation status (Supplementary Table 1, DAD-IS, https://www.fao.org/dad-is/browse-by-country-and-species/en/), we combined the high-definition network visualisation (NetView) and the model-based clustering results (ADMIXTURE) with the identification of “key contributors,” following the integrated three-step approach (Steinig et al., 2016). Steinig and collaborators define “key contributors” as individuals retaining the largest variation in the relevant genetic relationship structure within populations (the most informative). We ran the NetView analysis with 10,000 iterations to calculate the genetic contribution score (GCS). To further investigate phylogenetic relationships among the sheep breeds in our study, a network-building method (NeighbourNet) analysis based on the matrix of Reynolds’ unweighted distances previously calculated was first performed using SplitsTree v5.3.0 software (Huson and Bryant, 2006). NeighbourNet is a method for clustering taxa into hierarchically nested sets, similar to cladistics and Bayesian inference. However, it differs in that it does not strictly adhere to a branching model of descent. Instead, it allows for overlapping and intersecting sets, making it better suited for detecting conflicting signals in datasets, such as those resulting from borrowing and blending among evolutionary lineages. The key features of the method are as follows: i) relationships among taxa are inferred by calculating pairwise distances based on character data; ii) weighted splits are generated and combined using an agglomerative clustering algorithm; and iii) results are represented as a “split graph” or network diagram with a “tree-like structure” (consistent splits form a branching and tree-like appearance or “box-like structures”; inconsistent splits produce a latticed appearance, reflecting reticulation (blending)). More specifically, a network-like topology is a graphical representation that reflects relationships among populations, breeds, or taxa that go beyond a simple tree-like structure. Unlike a strictly branching phylogenetic tree, which assumes a single lineage of descent, a network-like topology accounts for non-hierarchical relationships such as gene flow, admixture events, and conflicting signals (situations where genetic data suggest multiple possible relationships between populations). In a network-like topology, these complex relationships are represented as reticulations (interconnected “box-like” structures) in the graph. These reticulations visually indicate genetic blending, admixture, or shared ancestry.

The level of reticulation in the network was quantified using the Delta score and Q-residual score (Grey et al., 2010; Holland et al., 2002). The Delta score and Q-residual score are metrics used to assess conflicting signals within a network, where conflicting signals are calculated first. Both measures evaluate path length discrepancies among pairs of taxa within “quartets” (groups of four taxa) sampled from the network. Quartet scores range from 0 to 1, with values closer to 0 indicating more tree-like splits and values closer to 1 indicating greater reticulation, suggesting complex relationships and possible admixture in the latter case.

A more concise description of the historical demographic relationships between populations can be derived by constructing admixture graphs, which are phylogenetic trees that include admixture events such as splits and mergers among internal nodes (i.e., populations) in their topology (Patterson et al., 2012). AdmixtureBayes is a Python-based programme designed to generate, analyse, and visualise posterior samples of admixture graphs. It provides a method for a more comprehensive exploration: the MCMC framework allows for a thorough search of possible admixture graph topologies, often identifying correct structures where other methods may fail. In addition, it allows for the quantification of uncertainty by providing measures of statistical uncertainty, reporting posterior probabilities of specific admixture events, and offering insights into the confidence of inferred relationships. We constructed admixture graphs to infer and analyse gene flow and admixture events that occurred in the past, applying a Bayesian approach implemented in AdmixtureBayes (Nielsen et al., 2023), available at https://github.com/avaughn271/AdmixtureBayes. Although the programme can generate different types of trees, we considered only the consensus trees. These are trees formed by combining all nodes whose posterior probability of appearing in an admixture graph exceeds a certain threshold. Three posterior probability thresholds were chosen (75%, 90%, and 95%). Analyses were performed running three independent chains, checking for convergence following Nielsen et al. (2023), and discarding 20% of each chain as a burn-in. Each chain was extended until the convergence was reached. More specifically, during the second step of the procedure, the convergence of the MCMC sampler was assessed first using the analyzeSamples.py script implemented in the programme, followed by examining the trace plots of the chain or the Gelman–Rubin convergence diagnostics of parallel chains using the included R script EvaluateConvergence.R. Thus, we reran the MCMC procedure five times, increasing the number of iterations each time, for a total number of 5,000,000 iterations, until all three chains had reached the same stationary distribution. For the construction of the consensus graphs, we analysed only chain1 using the following setting: -mcmc_results chain1. csv--burn_in_fraction 0.30 --thinning_rate 40 --slow.

## 3 Results

### 3.1 Data

After merging the datasets, there were initially 582,101 markers and 1,338 animals. However, filtering for minor allele frequency, poor genotyping, and autosomal location reduced the number of markers to 35,688. Additional filtering for LD reduced the dataset to 31,010 markers. The average genotyping rate was 0.998. After applying additional filters for low genotyping quality, excess heterozygosity, and presence of close relatives, 763 animals remained for the subsequent, final analyses.

Geographic information, label acronyms, names of breeds, number of animals per breed before and after the filtering steps, and origin of datasets are summarised in Supplementary Table S1; a geographical map illustrating the sample location is shown in Supplementary Figure S1. The data comprise genotypes obtained from 38 breeds from 16 countries (one transboundary breed, with samples from two countries). There were 5 to 30 animals per breed.

### 3.2 Genomic diversity and relatedness among breeds

**FIGURE 1:**
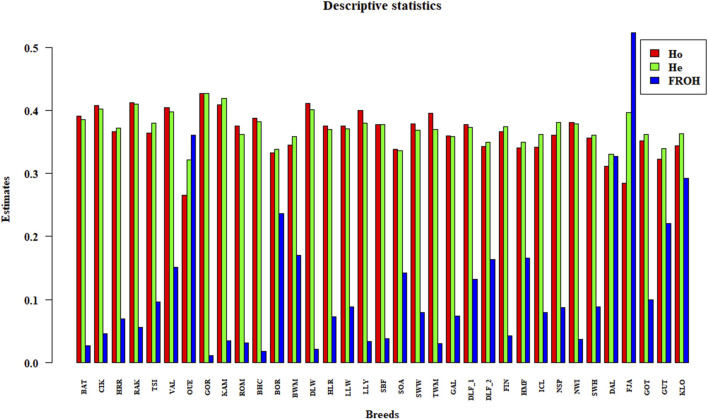
Histograms of descriptive statistics. The observed and expected heterozygosity (Ho and He) and genomic inbreeding (FROH) estimates for each breed are represented with vertical bars.

The pairwise F_ST_ matrix is shown as a heatmap in Supplementary Figure S2 and summarised in Supplementary Table S2. Among the Hungarian sheep, the two populations HRR and RAK are the most differentiated, with F_ST_ estimates of 0.09, compared to other Hungarian breeds. Meanwhile, BAT, CIK, and TSI are more similar to each other, and Merino has a slightly greater influence (F_ST_ = 0.05) on these last breeds compared to HRR and RAK (F_ST_ = 0.08). In contrast, VAL is the most distant member of the Eastern group. Comparing the distances between Hungarian breeds and the Northern group, estimates range from 0.07 to 0.1 with the Norwegian and Finnish breeds and from 0.1 to 0.17 with the Swedish breeds, whereas the Western group shows a range between 0.07 and 0.15. The observed (Ho) and expected heterozygosity (He) values and the genomic inbreeding coefficient (FROH) are shown in Supplementary Table S3 and Figure 1.

Within-breed levels of heterozygosity ranged from 0.36 to 0.4 for Ho and from 0.37 to 0.4 for He in the Hungarian breeds; considering all the breeds, OUE has the lowest values (Ho = 0.26 and He = 0.32), GOR has the highest (0.42 for both estimates), and the other breeds are in the range between 0.34 and 0.38 for both indices. Overall, the highest and lowest FROH values are 0.52 and 0.02, respectively. Specifically, the genomic inbreeding coefficient ranges from 0.045 to 0.09 for the Hungarian breed, with the exception of BAT (0.027). For the remaining breeds, the estimates are consistent with the previous studies (Beynon et al., 2015; Ciani et al., 2020; Rochus et al., 2020; Schönherz et al., 2020).

Exploring the distribution of ROH and the total coverage (sum of ROH or SROH) across the genome in each population allows us to quantify inbreeding levels and gain insights into aspects of populations’ demographic history. Following the distribution of length classes (Supplementary Figure S3), an initial survey revealed a common distribution pattern, with no ROH falling in the shortest class (0–2 Mb), and almost 50% of the homozygous segments in all breeds were between 4 and 8 Mb. In the Hungarian CIK and TSI breeds and Swedish FJA and KLO, almost 30% of segments are longer than 16 Mb. Analysing the SROH plot in Supplementary Figure S4 and focussing first on the Hungarian breeds, TSI is divided into two subgroups: one with individuals that have very long and more abundant ROH and another with individuals having a smaller fraction of the genome covered by ROH. BAT is the breed the least autozygosity, and the remaining breeds exhibit similar patterns (fewer and shorter ROH segments). Examining the other populations included in this study, some differences are discernible: NWI, FIN, TWM, DLW, BAT, ROM, and GOR breeds revealed the lowest number of autozygous segments in their respective groups (Northern, Western, and Eastern Europe). For ICL, SWH, NSP, SBF, GAL, and LLY breeds, we observed slightly longer and more ROH segments. FJA, BOR, and OUE have the greatest fraction of the genome covered by ROH.

### 3.3 Genetic structure and phylogeographic relationship

**FIGURE 2:**
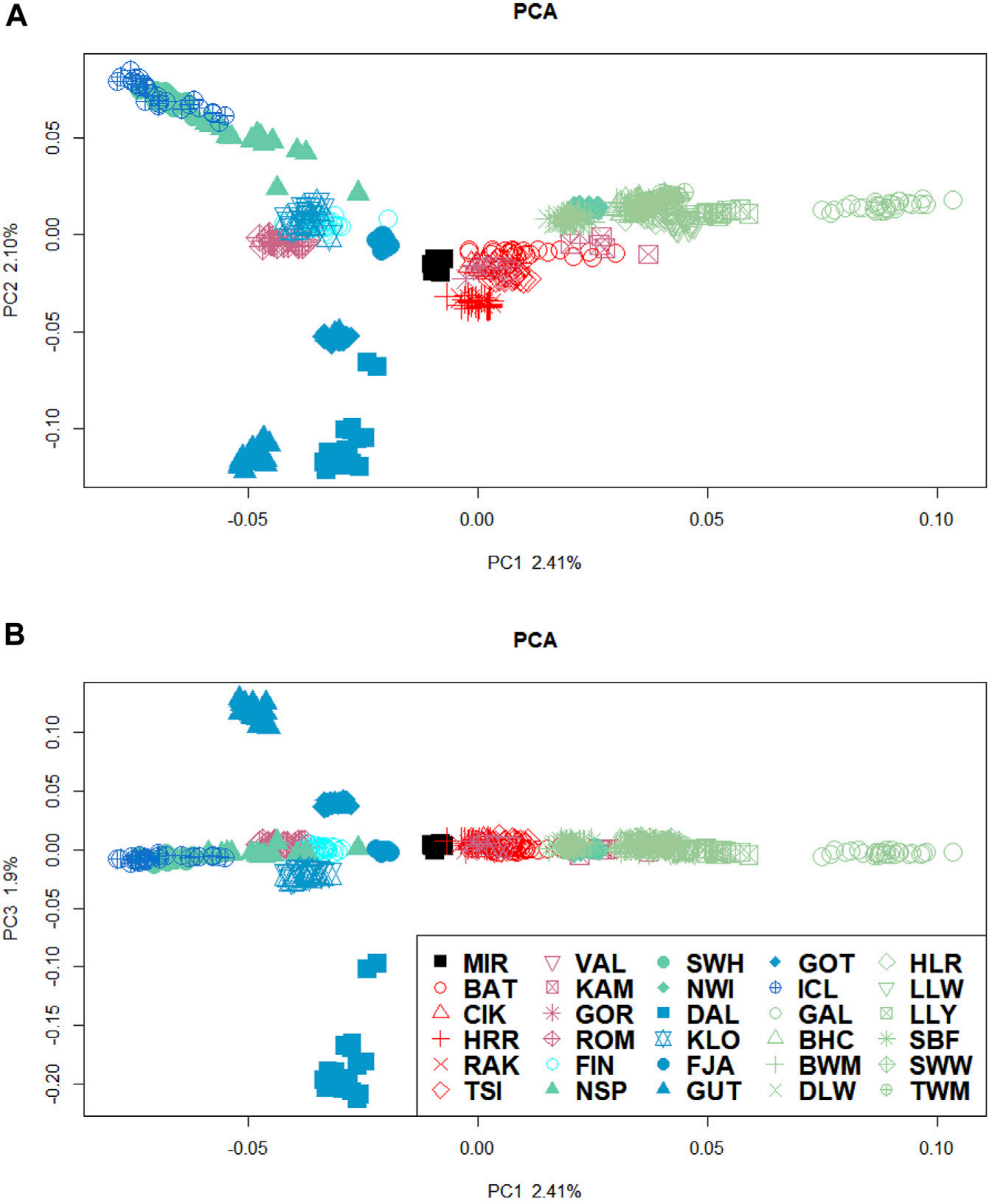
PCA of the dataset after excluding the most divergent breeds. **(A)** Scores for each individual for PC1–PC2 and **(B)** PC1–PC3. Colour of symbols indicates the country of origin (bright blue, Finland; blue, Sweden; green, Norway; darker blue, Iceland; light green, British Isles, red, Hungarian; dark red, remaining Eastern European breeds; black, *O. orientalis*. Symbols of the same colour indicate breeds within the country.

The clustering analyses allowed us to infer the population structure and individual genetic components. We performed the principal component analysis (PCA) twice: first with all the breeds included (Supplementary Figure S5) and then again with the most divergent breeds excluded to avoid large-sample bias and provide a better representation of their relationship. Figures 2A, B shows the PCA for the first three PCs.

The total percentage of variance accounted for by the first three PCs is almost 7%. The first PC (PC1) divides the breeds on the basis of the types of tails (short- and long-tailed sheep), with the ancestral MIR significantly closer to the Swedish populations and the Icelandic breed constantly clustering with the Norwegian group. The second component (PC2) distinguishes the Swedish (except KLO), Hungarian (CIK, RAK, and HRR), and Central–Eastern European (VAL and GOR) breeds from Norwegian, Icelandic, and the British Isles sheep. The third PC (PC3) distinguishes the Swedish populations DAL and GUT from the remaining Swedish groups.

**FIGURE 3:**
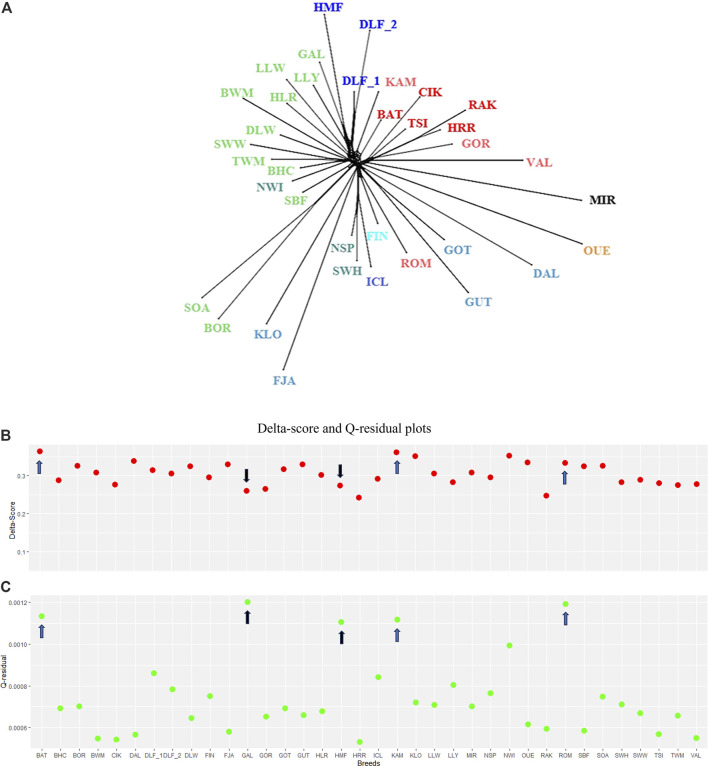
Phylogeographic inference. **(A)** NeighbourNet graph for the target breeds in our study based on Reynolds’ distance matrix. The branches corresponding to Northern, Western, and Central–Eastern Europe are indicated in blue, green, and red, respectively, within different shades, according to their geographical origin. **(B)** Plot of Delta scores (red) and **(C)** Q-residual (green) plots. For the description of the sheep breeds, see Supplementary Table 1.

The connection among breeds depicted by the PCA was supported by the NeighbourNet analysis based on Reynolds’ distances and is shown in Figure 3.

**FIGURE 4:**
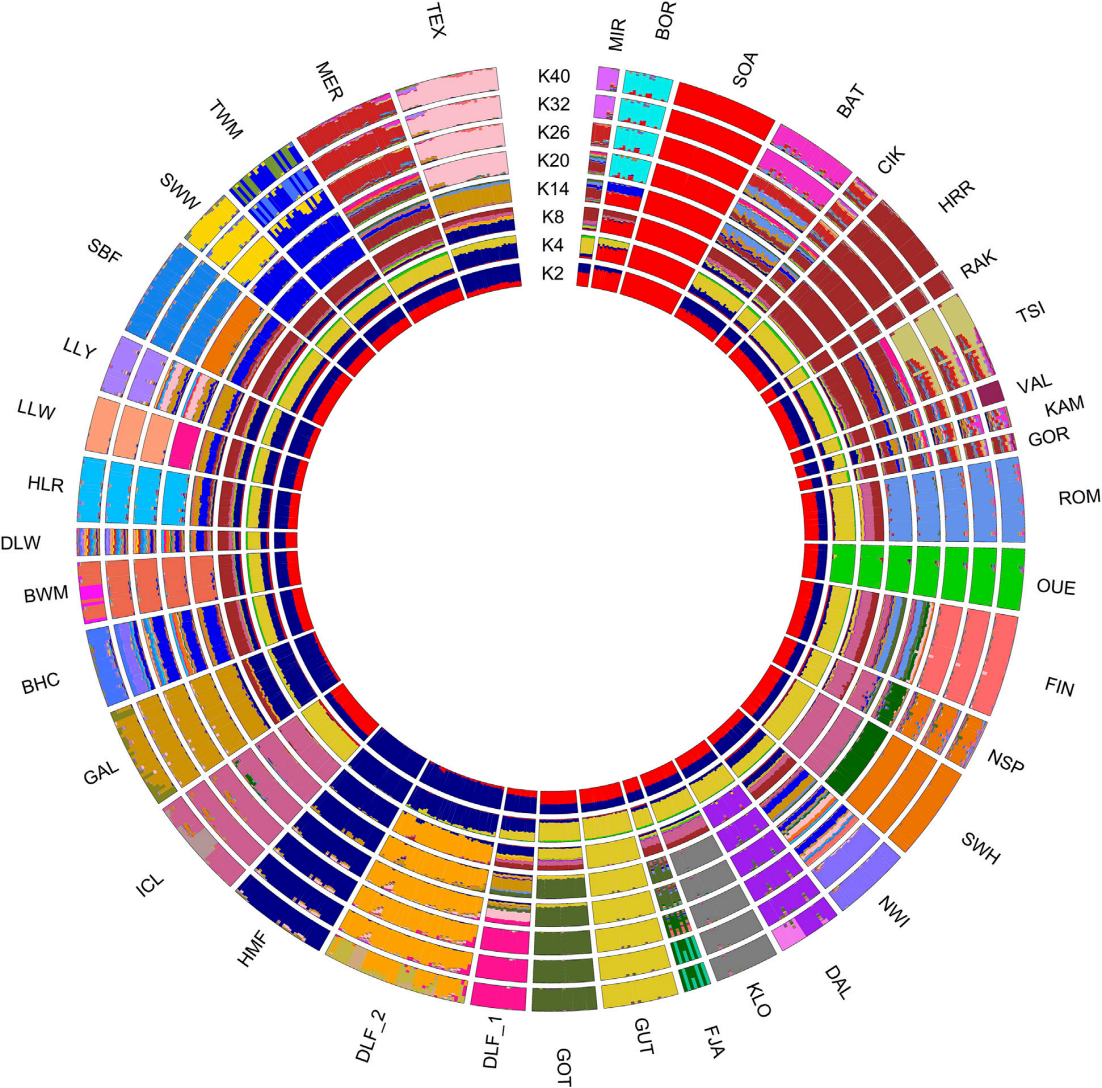
Admixture results summarised in a circular pattern for different values of K (number of clusters) increasing from the centre (K = 2) to periphery (K = 40). Different genetic contributions are shown as different colours corresponding to different genetic components. For the full definition of breeds, see Supplementary Table 1.

Despite the complexity of this exploratory analysis, the phylogenetic network (fit = 98.987, Figure 3A) approximately distinguishes the breeds by grouping the short- and long-tailed sheep. SOA, BOR, and SBF, archetypal of “primitive” breeds, are in a separate branch. The graph showed several clear clusters, but a closer inspection of the central part of the network revealed rectilinear webbing within clusters, with regions of conflicting signals for specific breeds. The Delta score and Q-residual score, which quantify the extent of reticulation for the whole network diagram and, thus, offer an accurate measure of departures from a strict tree, are summarised in Figures 3B, C. The estimates of the overall tree and non-tree-likeness of the network yielded an average Delta score of 0.3 and a Q-residual score of 7.457E-4. The highest scores belong to BAT and KAM (Delta score = 0.36 and Q-residual score = 0.001; blue arrows in Figures 3B, C). The measures of tree-likeness for HRR, RAK, GOR, and CIK are the lowest. An exception was the scores for Irish GAL and Danish HMF, where the Delta-score values were more tree-like, in contrast to the Q-residual values, which were slightly more network-like (Delta score = 0.26 and 0.27 and Q-residual score = 0.001; black arrows shown in Figures 3B, C). The results of the two metrics suggest a shift towards a slightly network-like topology, which is reflected in our phylogenetic reconstruction by the reticulated relationships that link the breeds analysed in this study. Individual admixture proportions were estimated using the model-based admixture clustering analysis and are shown in Figure 4.

The most probable number of K clusters (individual ancestry estimations) is 31 (Supplementary Figure S6), which is the estimation that exhibits the lowest log-likelihood value after applying the cross-validation method. In Supplementary Table S4, we reported the admixture proportions for the Hungarian breeds analysed in this study. We also evaluated the model fit using evalAdmix as the correlation of residuals (Supplementary Figure S6), and in our analysis, the residuals are uncorrelated among individuals, indicating a good model fit.

Overall, the analysis revealed that OUE, SOA, GUT, and HMF breeds maintained a distinct genetic profile compared to other European breeds. With K = 2, all the Hungarian breeds show both genetic components, as do the remaining breeds, except for SOA (red) and HMF (blue), which show only one component. At K = 4, the yellow component (most prominent in the Swedish GUT breed) was dominant in all populations, excluding the Danish breeds Galway, Ouessant, and Soay. At K = 8, the indigenous component of the Hungarian HRR is observed in all the Eastern European breeds, and from K = 20 onward, many breeds appear genetically well-characterised. Another noticeable consideration is the separation at K26 of TSI in two subpopulations, which is consistent with SROH analysis. Finally, at K = 32, all the breeds show a defined genetic identity except for some Eastern European (CIK, VAL, KAM, and GOR) and a few British Isles populations (BHC, DLW, and TWM).

**FIGURE 5:**
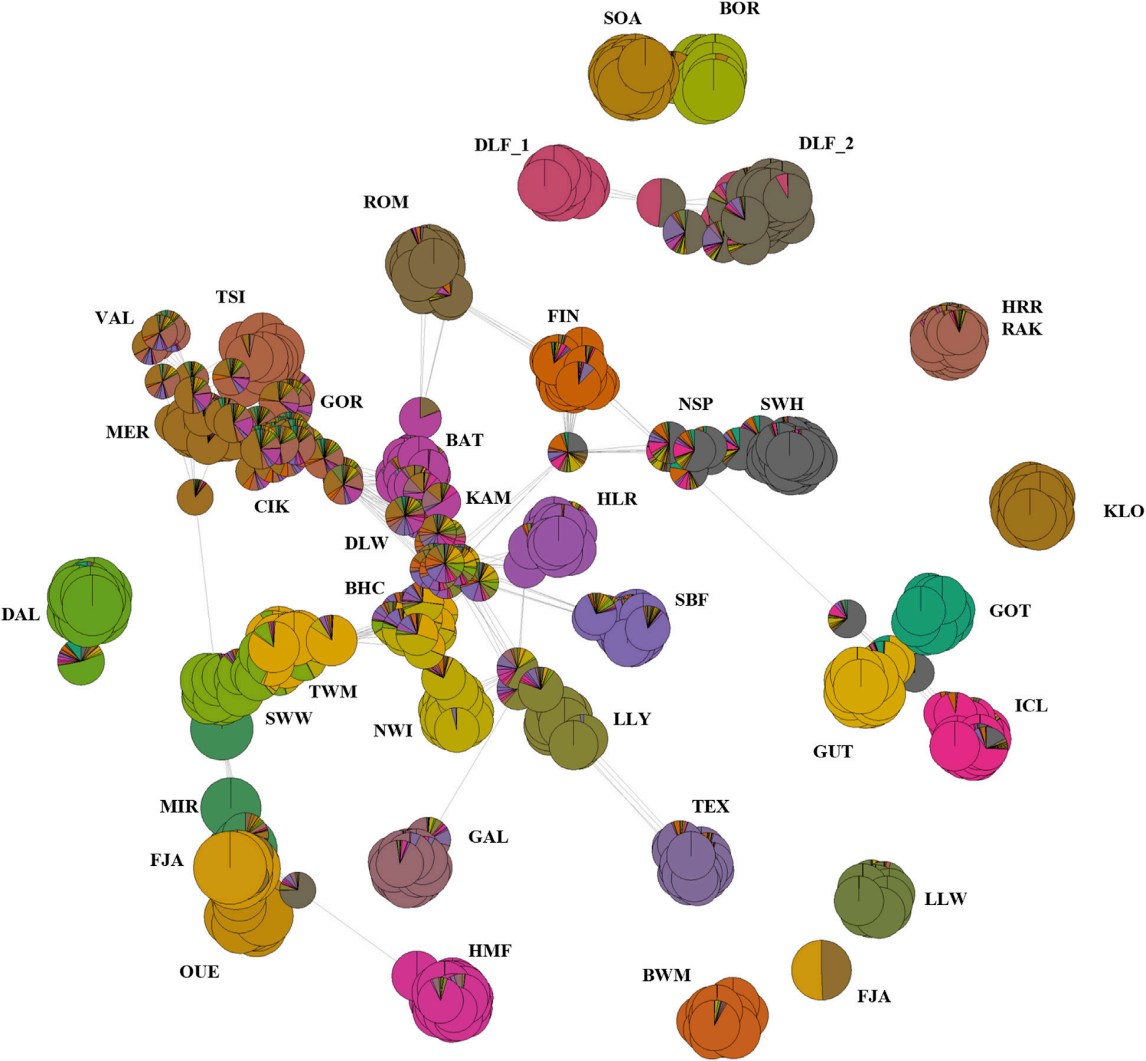
Network representing the genetic relationship among breeds and the key contributors. The network was built combining the results of the GCS (the genetic contribution of individuals within population) analysis and the estimates of individual admixture proportions (ancestries). We plotted the results using the most probable number of clusters (K = 31, optimal K-value selected by the cross-validation method in Admixture). Every circle or node represents one individual, and the breed code is indicated within each circle. The size of the nodes is proportional to its GCS reported in Supplementary Table 5. The grey lines are the edges that connect individuals in the network, and their width is proportional to the genetic distance between individuals. Breed codes are shown in Supplementary Table 1.

Since our analyses revealed a complex population structure, we applied a deeper analytical method that allows for the identification of high-resolution population structure and sub-structure. As shown in Supplementary Figure S7, the three methods concur in detecting k = 8 as the starting point to evaluate the relationship between populations. Starting from the larger values of k (Supplementary Figure S7A, k = 45), we observed the connection of more distantly related samples, with some relationships emerging according to the model-based clustering analysis. The Hungarian breeds are closely related to all the Eastern European breeds, with the exception of ROM, which is linked to the Finnish and the short-tailed breeds instead. The Swedish GOT, GUT, and DAL breeds appear the most divergent, together with the primitive BOR and SOA. For k = 33 (Supplementary Figure S7B), the indigenous HRR and RAK breeds are now distinguished from the remaining Hungarian breeds. We also detect more outliers and divergent groups across the short-tailed sheep. Further substructures within both short- and long-tailed sheep are evident (Supplementary Figure S7C, k = 20), with Eastern European populations still showing some relationship to each other, Merino, and two Welsh populations. Moreover, there were some isolated individuals with no connection at the periphery of the graph. Finally, at k = 8 (Supplementary Figure S7D), we can observe a finer-scale genetic structure; in particular, HRR, RAK, and ICL breeds are divided into two groups, revealing a strong substructure. As a complementary analysis, we combined the network information with the individual admixture proportions specified by the ADMIXTURE analysis (Figure 5). This approach provided more detailed information and allowed for the identification of individuals that could be defined as “key contributors.”

The size of the circle or node (that represents one individual with its admixture proportion) is proportional to the individual genetic contribution to such population substructure. If we focus on the breeds with a greater proportion of admixture (e.g., VAL and DLW), no key contributors are identified (each animal exhibits a little contribution). The opposite occurs for the Swedish breed (e.g., DAL and FJA), with some individuals ranked amongst the top key contributors (Supplementary Table S5). The topology of the network additionally illustrates the slightly lower impact of admixed sheep on the formation of some populations (SBF, LLY, SSW, TWM, BWM, and ROM). Another observation is the high proportion of Merino-like ancestry in all the Eastern European breeds, except Tsigai, the two populations of Hungarian Hortobagyi Racka, and Romanov. Although this analysis has some limitations in terms of intuitiveness, as it is a two-dimensional (2D) representation of a three-dimensional 3D model, it still allowed us to identify some well-known relationships among our breeds such as BAT, FIN, and ROM. However, there are some noticeable differences compared with the admixture analysis.

One more important aspect in the investigation of breed origin and formation is observing the fluctuation in the effective population size over time through the assessment of the historical $N_{e}$ estimates. Supplementary Figure S9 illustrates the $N_{e}$ trend for the target breeds (in A, Hungarian breeds from 1 to 600 generations; in B, with a focus on the last 100 generations; and in C, the entire Eastern European group). Although we calculated $N_{e}$ for the five Hungarian populations (summarised in Supplementary Table S6, considering the last 10 generations, which reflect the last 20–30 years), we plotted the decay curves excluding BAT as this local breed has only recently been created by crossbreeding. Exploring the curves and assuming a generation time of 3 years for sheep (Miranda et al., 2023), we observe that approximately 500 generations ago, a gradual expansion started for the Tsigai breed. For the two populations of Hortobagyi Racka, this expansion started approximately 300 generations ago, while Cikta experienced a very significant increase in $N_{e}$ during the same timeframe. The curve for CIK begins to decline approximately 100 generations ago (that is circa 300 years ago), suggesting a strong reduction in its population size (see also Supplementary Figure S9B). However, the remaining Hungarian breeds exhibit a gradual decay with a stable, low population size. The decline in population size for Hungarian breeds started approximately 120–40 years ago due to historical bottlenecks, with a more recent reduction in $N_{e}$ for TSI and CIK. When we consider all the Eastern breeds (Supplementary Figure 9C), GOR, KAM, and VAL show growth in their population size between 200 and 100 generations ago, followed by a sudden decrease in the most recent generations (approximately 100 years ago). To complete the picture of the historical relationships between populations, we performed a Bayesian analysis that provides more information on breeds of mixed origin.

**FIGURE 6:**
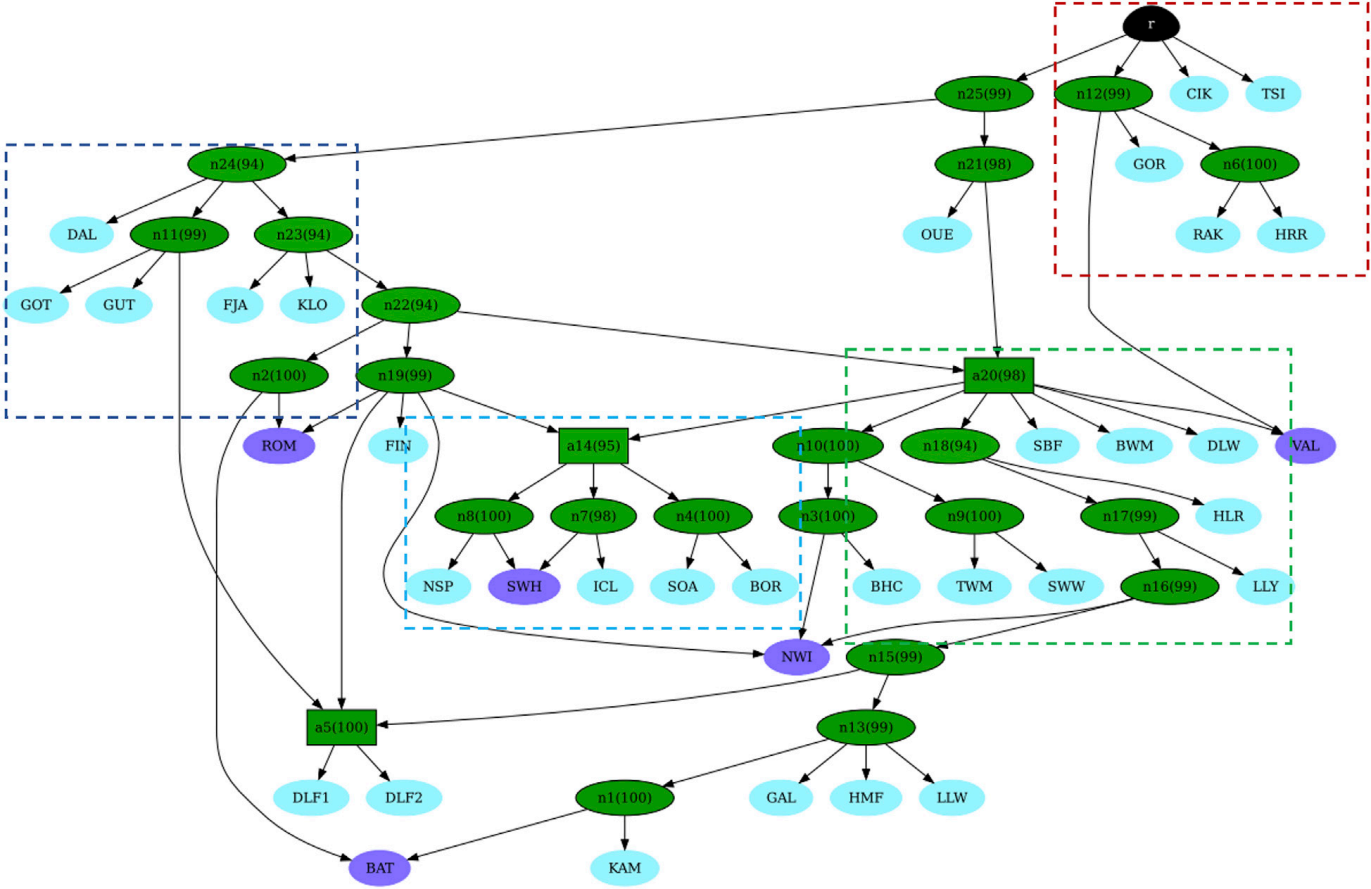
Consensus admixture graph at 90% of posterior probability as inferred by AdmixtureBayes. Each leaf corresponds to a population/breed (breed codes as shown in Supplementary Table 1). Leaves, which are the product of an admixture event, are shown in purple. Otherwise, the leaves are light blue. Internal nodes are green. Shades of green show the posterior probability (numbers in parentheses) of the true graph having a node with the same descendants. Darker shades of green correspond to higher probabilities. The root is shown in black.

After checking for convergence (Supplementary Figure S10), we built the graph. Figure 6 illustrates the consensus admixture graph at 90% posterior probability. In this graph, where the branch length is proportional to the distance among breeds, four main clusters can be identified, each labelled within dashed rectangles. The first cluster, shown in red, represents the Eastern European breeds. This cluster is the closest to the root and well-separated from the others (except Ouessant, OUE). The second cluster, shown in dark blue corresponds to the Swedish cluster; this cluster is connected to the root via two distant internal nodes (n25 and n24). The internal node where the OUE branches off (n21) connects to the whole British Isles group (third cluster, in green). A more internal, intermediate node (a14), which links both the second and third clusters, connects them to a fourth cluster (in light blue), which is represented by the Norwegian samples. Interestingly, the same node (a14) gives rise to the Icelandic and the primitive Scottish breeds, which appear to share a common ancestry. Separated by long branches and located at the end of the admixture graph, we retrieve the Danish populations. Figure 6 shows the admixture graph, which provides more information, visibly highlighting the different derivations of TSI, CIK, and HRR/RAK (red dashed square) and the ancient origin of the French OUE, followed by the Swedish DAL. In our graph, the position also suggests its role as the “ancient contributor” between the Nordic and the Western genetic components and a cryptic proximity to the Hungarian and the Polish breed GOR; this outcome should be interpreted with caution. In addition, while some admixture events (highlighted in purple in Figure 6) indicate recent introgressions (e.g., BAT and NWI), others are not documented or previously detected (e.g., SWH). Moreover, the ROM breed showed “Nordic” genetic contributions from Sweden and Finland. Supplementary Materialsl (datasheets 1 and 2) contains the consensus admixture graphs obtained at 75% (datasheet 1) and 95% (datasheet 2) posterior probability, which identify the same admixed populations.

## 4 Discussion and conclusion

By integrating a multidisciplinary approach that connects archaeological findings, zooarchaeological remains, historical written sources, and modern genomic data, researchers can explore a broader range of aspects related to sheep domestication. This comprehensive approach allows for a deeper understanding of the intricate web of interactions and migrations between different populations and the underlying forces that prompted these movements. For instance, one such driving factor could be production objectives, as observed in the spread of woollier sheep breeds during the revolution of secondary products, when sheep began to be valued not only for meat but also for wool. This kind of analysis provides valuable insights into the complex processes and societal influences that shaped the development and movement of sheep populations across various regions and time periods (Macheridis, 2022; Sabatini et al., 2019). However, despite these advancements, the demographic history of sheep, particularly European breeds, remains an ongoing area of study, with uncertainties surrounding the precise timing and nature of key events. For instance, questions still remain about the origin and divergence of haplotypes, the distribution of various lineages, and whether the gene flow between populations was bidirectional or unidirectional (Miranda et al., 2023). This complexity is especially pronounced in the regions of Central and Eastern Europe, which have long served as a bridge between Europe and Asia. These areas have been shaped by the convergence of numerous cultures, economies, and trading networks and have seen the development and decline of empires, wars, and migrations over centuries. Such a rich historical backdrop has left a profound mark on the genetic landscape of sheep in these regions, making it an essential focal point for understanding the broader patterns of domestication and breed evolution. Therefore, in the present study, our main objective was to genetically characterise four indigenous Hungarian breeds, one of which was recently created, while the others have a more ancient background and complex ancestry, by analysing their population structure and admixture, as well as their relatedness to neighbouring breeds. Since Hungary has been the convergence point of different expansion and trading routes (such as “the Varangians to the Greek,” a mediaeval trade route that connected Scandinavia, Kievan Rus, and the Eastern Roman Empire), we explored the pattern of admixture, searching for some trace of Hungarian ancestry in Scandinavian sheep or *vice versa*. Hungary’s central position in these historical networks makes it a key region for studying potential genetic exchanges between distant populations.

Our results on descriptive statistics (heterozygosities, fixation index, and genomic inbreeding coefficient) for the dataset including all breeds align with previous SNP array studies. Hungarian sheep populations have predominantly been studied using mitochondrial DNA, cytochrome B and D-loop regions, and microsatellite markers (Gáspárdy et al., 2021; 2022; Kusza et al., 2008; 2011). Although research on SNPs to analyse the diversity of Hungarian indigenous sheep has been limited, with one notable study by Zsolnai et al. (2021), further publications on this topic are currently in progress. Zsolnai and colleagues (2021) examined the genetic status of lowland-type Racka sheep in both black and white colour variants. Our results from the ROH analysis for the two populations closely align with those reported for the white variant by Zsolnai et al. (2021). Additionally, their study highlighted a severe population bottleneck in the breed following World War II, with gradual recovery beginning after 1983, largely due to efforts by the association of Hungarian Racka breeders (Veress et al., 2002). Our historical analysis of $N_{e}$ indicates that the Hortobagyi Racka (HRR) sheep experienced a significant population decline approximately 35 generations ago, which corresponds to roughly 70–100 years ago when considering a generation time of 2–3 years. In contrast, the decline observed in the Racka (RAK) population is more recent, occurring approximately 15 generations ago or 40 years ago. This historical bottleneck is further corroborated by our assessment of the total coverage of homozygous segments within these two populations. Although our $N_{e}$ estimates are approximate due to the limitation of the dataset or variations in the assumed generation time for sheep (Miranda et al., 2023), they align with documented demographic changes observed in the populations. Additionally, it is important to note that effective population size estimates derived from linkage disequilibrium may vary, particularly in the context of significant migration events, which are relevant to our study (Novo et al., 2023; Ryman et al., 2019). Another critical factor that may influence $N_{e}$ estimates is the population structure itself. Simulations conducted by Novo and colleagues (2023) have illustrated a recurrent pattern of $N_{e}$ reduction over time, which can manifest either as a sharp decline in the most recent generations or as a gradual linear decrease. Our findings may reflect this trend as some levels of substructure have been identified through NetView analysis. Consequently, results that exhibit these patterns should be interpreted with caution and warrant further in-depth demographic analysis to fully understand their implications. CIK and TSI have longer ROHs, suggesting a slightly more recent inbreeding. Kovács et al. (2019) reported that the Cikta stock of the early 21st century comprised genetically related individuals, following a bottleneck that happened around 1970, which supports the trend of historical $N_{e}$ (Supplementary Figure S9B). In the same study by Kovács et al. (2019), based on microsatellites, the authors compared Cikta and Tsigai breeds and discovered a more pronounced genetic homogeneity in Tsigai, a result that is noticeable in our NetView analysis incorporating the admixture results. Gáspárdy et al. (2021), Gáspárdy et al. (2022) analysed two mitochondrial sequences (mtDNA control regions CR and CytB) and obtained similar results, with Tsigai consisting almost exclusively of slightly differentiated individuals. They also obtained a unimodal mismatch distribution for Tsigai and a bimodal distribution for Cikta, generally associated with a sudden expansion and a more constant population size, respectively. In addition, the bimodal peaks suggest the occurrence of two distinct sub-populations (Alvarado Bremer et al., 2005). Furthermore, the complexity of the formation of the Cikta breed is also reflected in the NetView graph, where many individuals exhibit genetic components similar to Merino, Finnish, Norwegian, and English breeds. This complexity highlights the diverse genetic influences that have shaped the Cikta breed throughout its development. This observation aligns well with the breed’s maternal lineage, shaped by several migratory events, which resulted in the presence of three haplotypes (A, B, and C). In contrast, the indigenous Tsigai and Hortobagyi Racka breeds primarily show haplotype B (of European origin) and, to a lesser extent, haplotype A (from Eurasia) (Gáspárdy et al., 2022). The occurrence of haplotype C in Cikta can be attributed to the following three key events: i) approximately 6500 BCE, when shepherds from the Altai cultural groups and their sheep reached the southern Carpathians; ii) during the Iron Age (approximately 560 BCE), with the arrival of the Scythians in present-day Hungary; and iii) during the Ottoman Empire (16th–17th centuries), when fat-tailed sheep—likely carriers of haplogroup C—were integrated into local populations (Gáspárdy et al., 2022).

The significant reduction in the population size among Hungarian breeds and the introgression of foreign alleles is a situation common to many traditional breeds worldwide. This decline has primarily been driven by changes in breeding objectives and the need for higher production to meet increasing demand (Sabatini et al., 2019). Additionally, historical events such as the World Wars have further exacerbated this decline in Hungary, leading to a lasting impact on local sheep and other livestock populations. As a result, several Hungarian breeds, historically exploited for triple purposes and widespread across Europe since the early Middle Ages, were unable to compete with more productive British and Southern European breeds and were gradually replaced (Bodó, 1994; Deng et al., 2020; Gáspárdy et al., 2021). The preservation of these breeds now depends on non-commercial initiatives, with their survival maintained by small, fragmented populations in a few flocks. This situation contributes to the high levels of inbreeding detected in this study and previous studies. Specifically, the analysis of both contemporary and historical population sizes and the distribution of ROH across length classes for the Cikta (CIK) and Tsigai (TSI) breeds suggest a decrease in population size and subsequent inbreeding, which is consistent with historical bottlenecks, a trend already observed in previous studies based on STR and mtDNA data (Gáspárdy et al., 2021; 2022; Kovács et al., 2019). Recent or past demographic events, insufficient resolution due to the limitation of the dataset, and other factors can obscure signals of the gene flow. However, several interesting findings emerged from the admixture graph (Figure 6). First, the most striking result is the distinct separation of both studied Hortobagyi Racka populations from other Hungarian and Eastern European breeds, as supported by cluster analyses, network analyses, and the admixture graph itself. This analysis also highlights the different origins of the Cikta and Tsigai breeds, which reflect their evolution from distinct sheep types. Second, the analysis suggested a more ancient origin for the Ouessant and Swedish Dalapäls breeds, while other Swedish breeds appear to have more recent origins. The breeds labelled in purple on the admixture graph are “breeds with mixed origins,” as identified by the analysis. For instance, the Bábolna Tetra (BAT) is a synthetic breed developed relatively recently through the crossbreeding of Merino sheep with the hardy Romanov and Finnish Landrace breeds (Neubauer et al., 2015). The Norwegian white Spaelsau (NWI) breed, officially formed in 2000, was developed through the crossbreeding of several Northern European breeds (Oliveira et al., 2020). The Valachian, an autochthonous breed from Slovakia, was crossbred with English mutton breeds in the mid-20th century to improve productivity while retaining rustic traits (Mészárosová et al., 2022). These mixed origins are well-documented, and the analysis successfully identified them. Notably, the Romanov population also appears to have a mixed origin, descending from a Swedish ancestor and sharing a common ancestor with the Finnsheep. This finding is consistent with historical records, which describe the Romanov as an independent branch of Northern European short-tailed breeds (Ivanov, 1935). The shared ancestry between Romanov, Norwegian Spaelsau, and Finnsheep has been suggested in earlier studies (Deniskova et al., 2018; Kijas et al., 2012; Ryder, 1983; Tapio et al., 2006). The Romanov breed, known for its extraordinary prolificacy, is the only short, thin-tailed breed in Russia and is bred globally for its valuable traits (Deniskova et al., 2018; Veniaminov, 1984). Although the programme is a robust tool to infer and visualise quite complex admixture histories in populations, the results have to be carefully interpreted by taking into consideration some limitations associated with our dataset and those associated with the programme itself. For example, the limited number of populations/breeds in the dataset is a balance between the newly genotyped data, the genotypes already available from publicly available databases that meet our requirements, and the computational effort, time consumption, and scalability issues of the inference. When the number of populations increases, the complexity of the admixture graph increases exponentially, making the process computationally more expensive, slow, and difficult to infer. Despite these limitations, the programme performed well by generating a topology that correctly identified admixed breeds when choosing a more stringent threshold for the posterior probability (Supplementary Material, datasheets 1 and 2). Overall, the analyses of individual genetic components, such as NeighbourNet, NetView, and AdmixtureBayes, highlight the gene flow between Hungarian long-tailed sheep and Nordic short-tailed sheep. The NeighbourNet and the associated metrics, which quantified the degree of reticulation of the network, allowed us to identify signals of genetic exchange that cannot be captured by simple tree-based models. These reticulations visually suggest genetic blending, admixture events, shared ancestry, and other evolutionary dynamics, enabling a deeper understanding of breed history and the genetic impact of interbreeding. However, its complexity requires a careful interpretation since assumptions about the nature and timing of the gene flow or other demographic events, such as population decline or expansion, may impact results. We detected a weak but identifiable Finnish and Norwegian genetic influence in the Cikta breed (Figure 5) and traces of the older Hungarian Racka in both Finnsheep and Romanov breeds (Figure 4, K = 8), suggesting bidirectional genetic exchanges between these populations. Investigating the paternal origins of several sheep breeds, Deng et al. (2020) identified migratory events responsible for the development of five lineages (y-HA, y-HB1 and 2, y-HC, and y-HD). Haplotype y-HC, present in Scandinavia, Western Russia, England, and at low frequencies in Spain, most likely spread early with primitive Northern European sheep (with a hair coat). A later wave, associated with y-HC-carrying English breeds specialised in meat production, became transboundary in the last five centuries (Meadows et al., 2004; Kijas et al., 2012). Archaeological evidence supports this expansion model, suggesting that fat-tailed sheep carrying haplotype y-HB spread approximately 3,400 years ago (Deng et al., 2020).

Based on archaeological and genetic evidence, Sabatini et al. (2019) proposed another wave of sheep expansion during the Bronze Age, introducing woollier animals from the Middle East to continental Europe. Százhalombatta-Földvár, located on the right side of the Danube approximately 30 km south of Budapest, is one of the largest temperate settlements from the Bronze Age in Central Europe. The sheep remains discovered at this archaeological site, dated to 1500 BCE, indicate that Hungary was among the earliest regions for fine wool production. This finding could explain the presence of Hungarian alleles in some short-tailed sheep breeds, possibly resulting from the introduction of new, woollier animals. On the other hand, the Nordic genetic influence in Cikta may reflect an ancient gene flow, possibly linked to Varangian migrations across Eastern Europe. Another possibility is a more recent introgression of Finnsheep, a breed commonly used to enhance fertility in others (Branford Oltenacu and Boylan, 1981), although there is no recorded use of Finnsheep in Cikta breeding. Although these genetic remnants linked to human activity may be obscured by crossbreeding, bottlenecks, and selection (Gopalan et al., 2022), the study of human–animal relationships continues to shed light on unresolved historical events (Manunza et al., 2023; Miranda et al., 2023).

This study exemplifies the challenges of dating specific genetic events, such as bidirectional gene flow, admixture, and haplotype divergence, as well as the difficulty in providing a historical context for these findings. The lack of written documentation for certain historical periods often obscures the origins and evolution of local breeds. This is particularly true for Hungary’s autochthonous Hortobagyi Racka sheep, whose origins remain unknown due to limited historical records, especially during the Turkish occupation (1,552–1,693) (Gáspárdy et al., 2022, Yunusbayev et al., 2015). Overall, our results not only offer new insights into the history, evolution, and relationships of Hungarian breeds with neighbouring populations, but they also raise further questions about the cultural, political, and biological processes that have shaped human societies. New discoveries challenge certain historical assumptions, particularly regarding the influence of Scandinavians (mainly Varangians) on the formation of the Hungarian nation and their cultural exchanges with the Magyars, ancestors of present-day Hungarians. The Varangians–warriors, traders, and settlers primarily from present-day Sweden–also ventured into Russia, Ukraine, and the Byzantine Empire from the 10th to 14th centuries (Gleason, 2009; Mägi, 2018; Price et al., 2019). It is believed that these Varangians spread northern sheep breeds during their travels. Excavations at the Viking Age settlement of Birka in southeastern Sweden have uncovered artefacts of possible Magyar origin, suggesting deeper connections (Benedict, 2009; Hedenstierna-Jonson, 2009). Additionally, Hungarian coins found in Viking Age contexts in Eastern Scandinavia hint at possible trading contacts, suggesting a Varangian presence in Magyar territory (Tentiuc, 2020). Combined evidence from graves, settlement layers, folk traditions, and linguistic studies (Moravcsik, 1946; Hedenstierna-Jonson, 2009; 2012; Mägi, 2018) suggests that Magyar–Varangian cultural exchanges went beyond mere trade or warfare.

In the 9th century, the Varangians migrated to Eastern Europe and established Kievan Rus, an early East Slavic state. The term “Rus” is considered to derive from the Varangians, highlighting their significant role in its foundation. As the Rus integrated with local Slavic populations, they developed a unique cultural and political identity. They controlled vital trade routes connecting Scandinavia to the Byzantine Empire, facilitating economic exchange and cultural interactions with neighbouring regions, including Hungary. During the 10th and 11th centuries, the Hungarians were also forming their identity in the Carpathian Basin. The arrival of the Hungarians coincided with Rus’s expansion, leading to potential interactions. Overall, the connections between the Varangians, the Rus, and early Hungarian rulers underscore a complex interplay of trade, diplomacy, and cultural exchange that was vital in shaping the identities of both the Rus and the emerging Hungarian state.

Our study aimed to provide historical context for these genetic findings by discovering clues such as introgression events that may correlate with large-scale human movements or mechanisms like trade, tribute, or the introduction of breeding stock. Although our analysis of four local Hungarian sheep breeds provides valuable insights, future studies incorporating the remaining native breeds would be crucial for understanding the complete genetic diversity of sheep in Hungary as these breeds reflect distinct historical migration patterns and adaptations in the Carpathian Basin. Moreover, although it is tempting to relate these findings to known historical events and the resulting assumptions, more in-depth analyses are needed to formally test our hypothesis, possibly exploiting ancient DNA information and using more refined methods to better understand the historical demography of the breeds under study.

## Data Availability

The datasets presented in this study can be found in online repositories. The names of the repository/repositories and accession number(s) can be found at: http://webserver.ibba.cnr.it/smarter/, https://doi.org/10.46471/gigabyte.139 (Cozzi et al., 2024).
