# Supplementary figures and images for: Exploring the complex population structure and admixture of four local Hungarian sheep breeds
Source: Front Genet. 2025 Mar 19;16:1507315. doi: 10.3389/fgene.2025.1507315 (PMC11962792; doi:10.3389/fgene.2025.1507315)

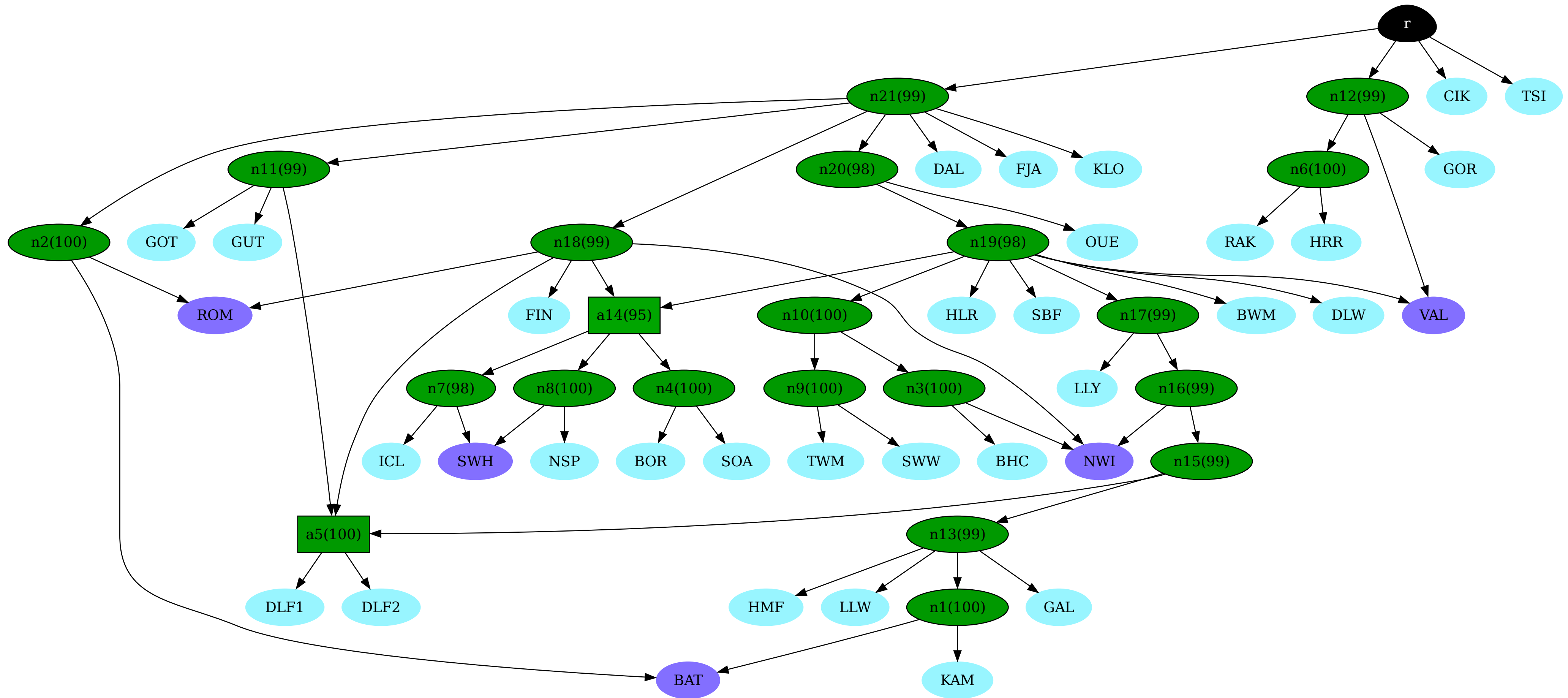

Supplement: Supplementary file 1 [file DataSheet2.pdf]

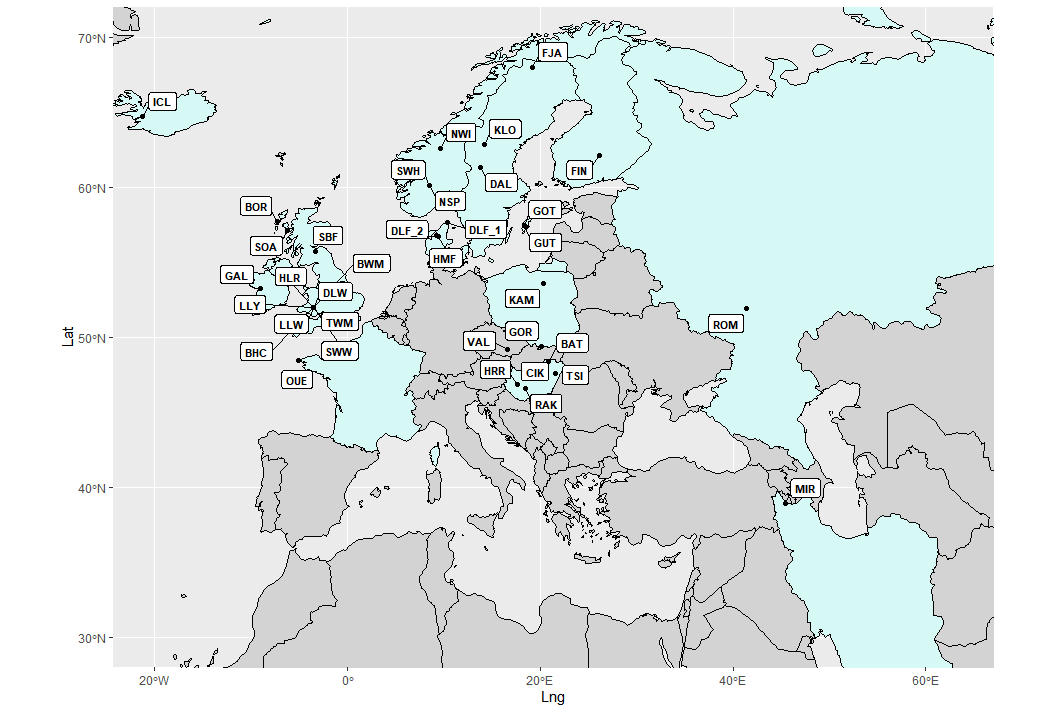

Supplement: Supplementary file 3 [file Image1.tiff]

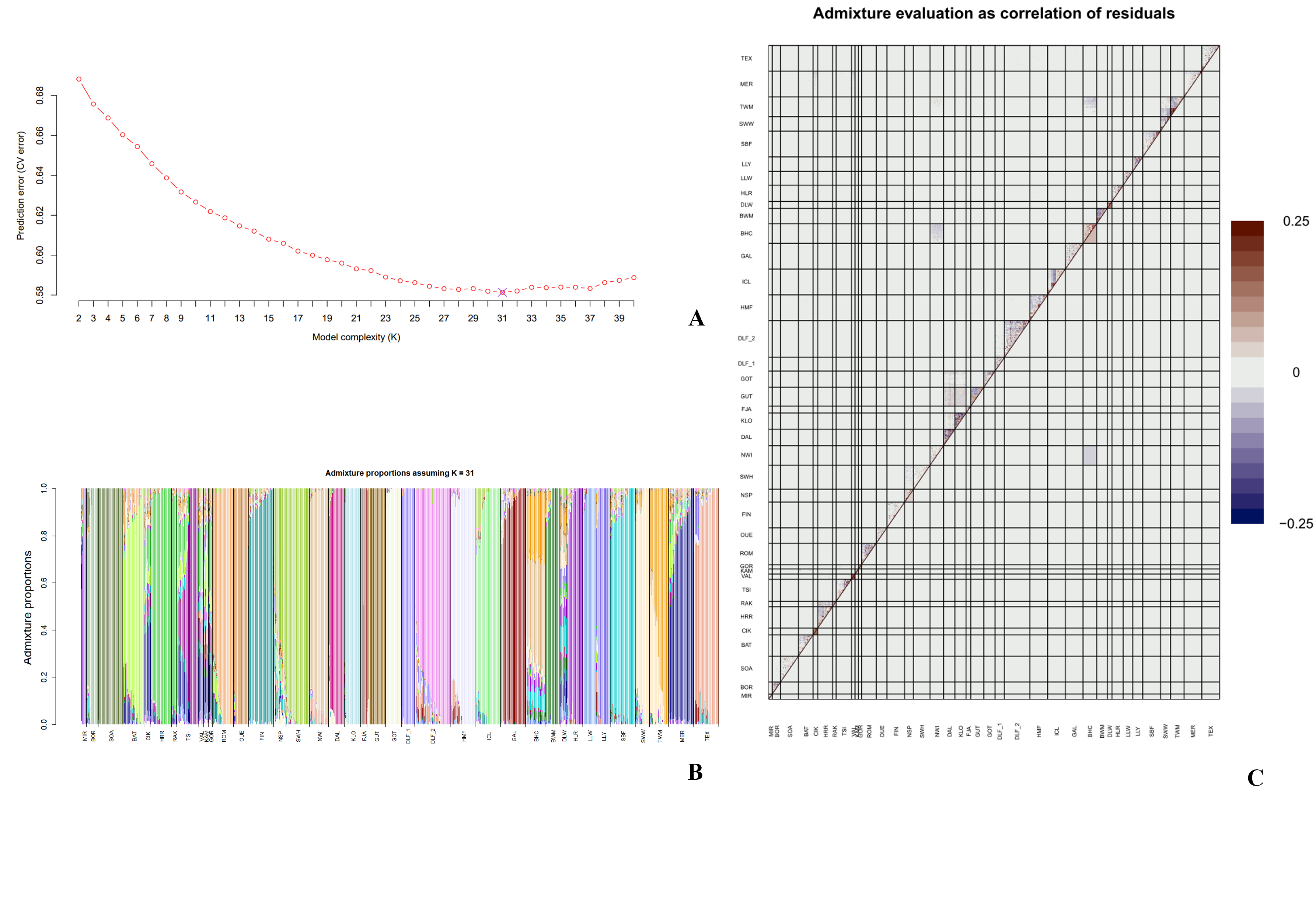

Supplement: Supplementary file 5 [file Image6.tif]

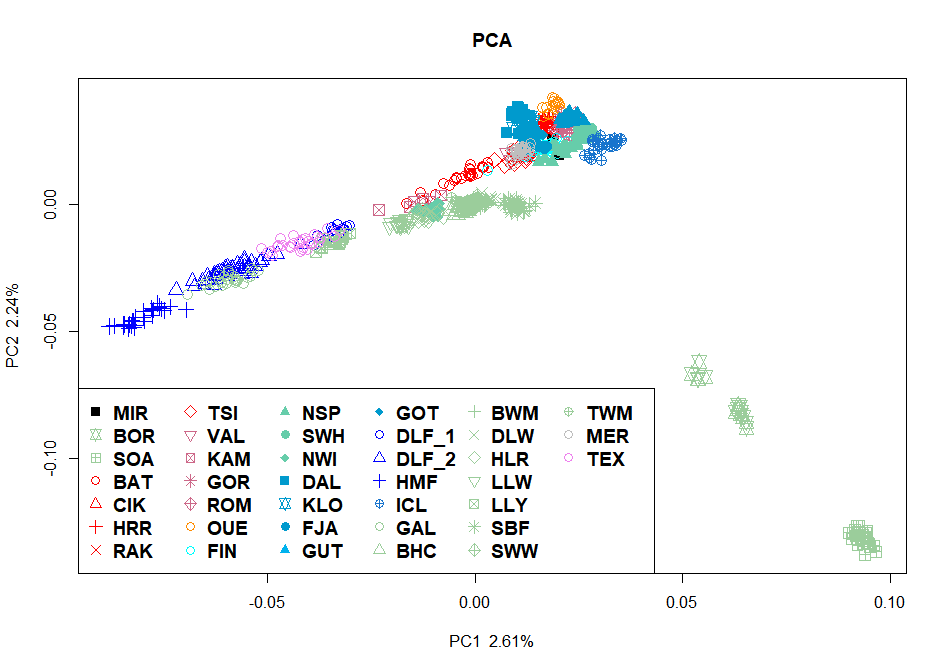

Supplement: Supplementary file 7 [file Image5.tiff]

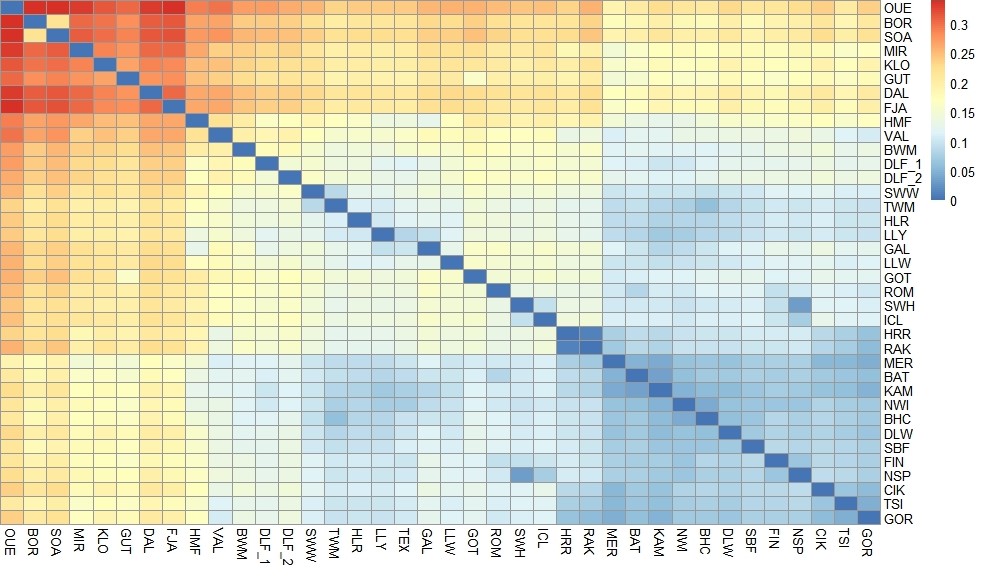

Supplement: Supplementary file 9 [file Image2.jpeg]

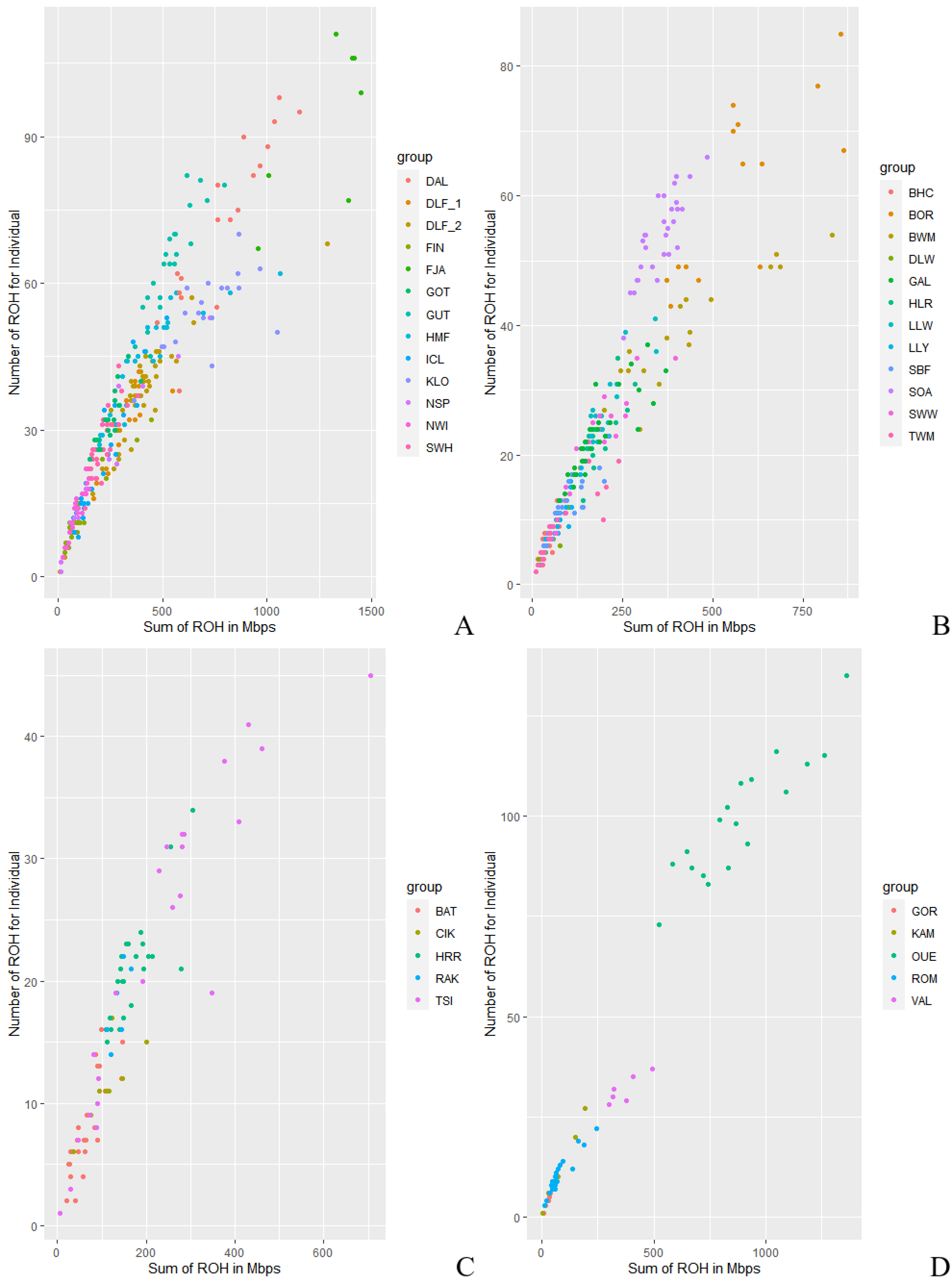

Supplement: Supplementary file 10 [file Image4.png]

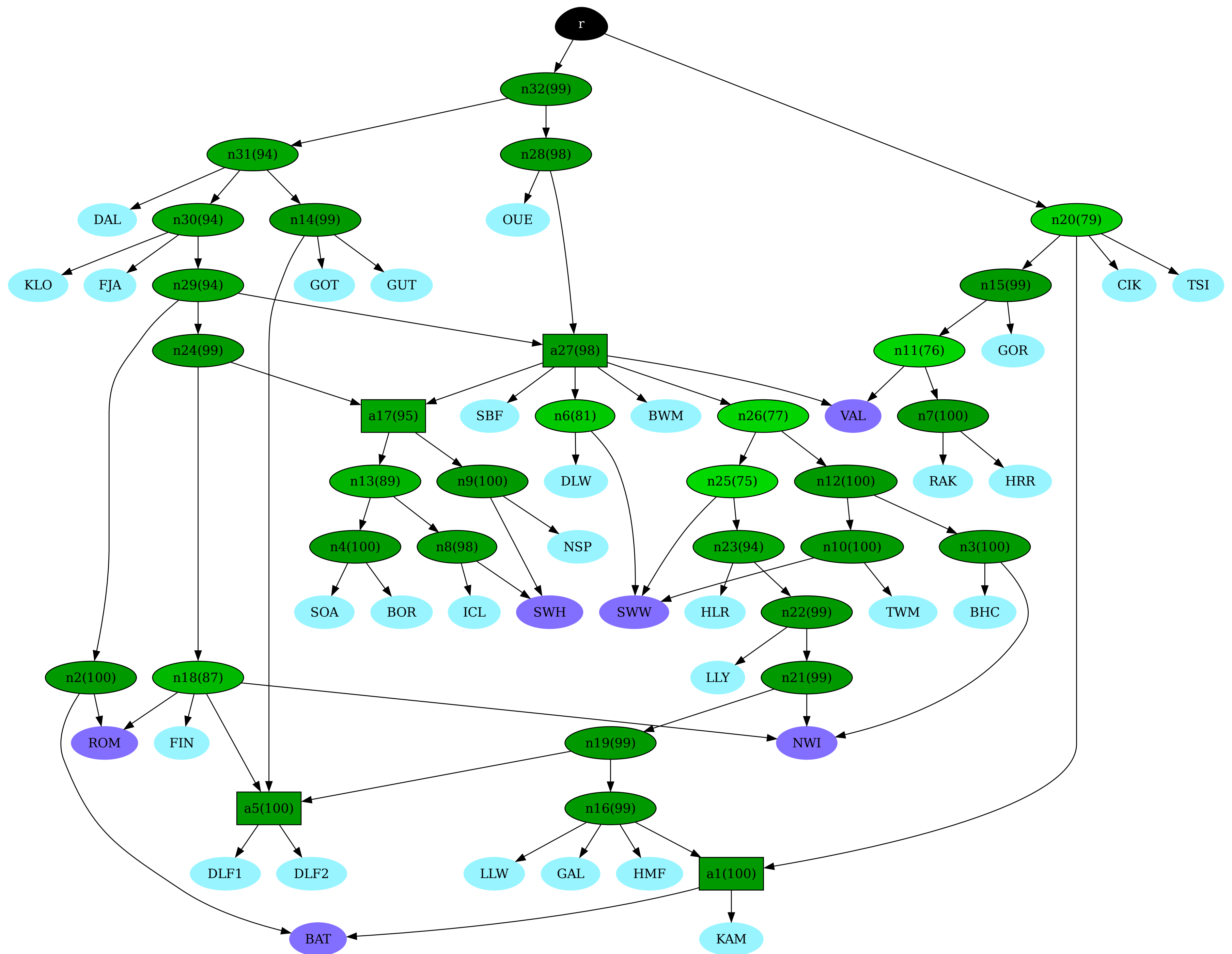

Supplement: Supplementary file 12 [file DataSheet1.pdf]

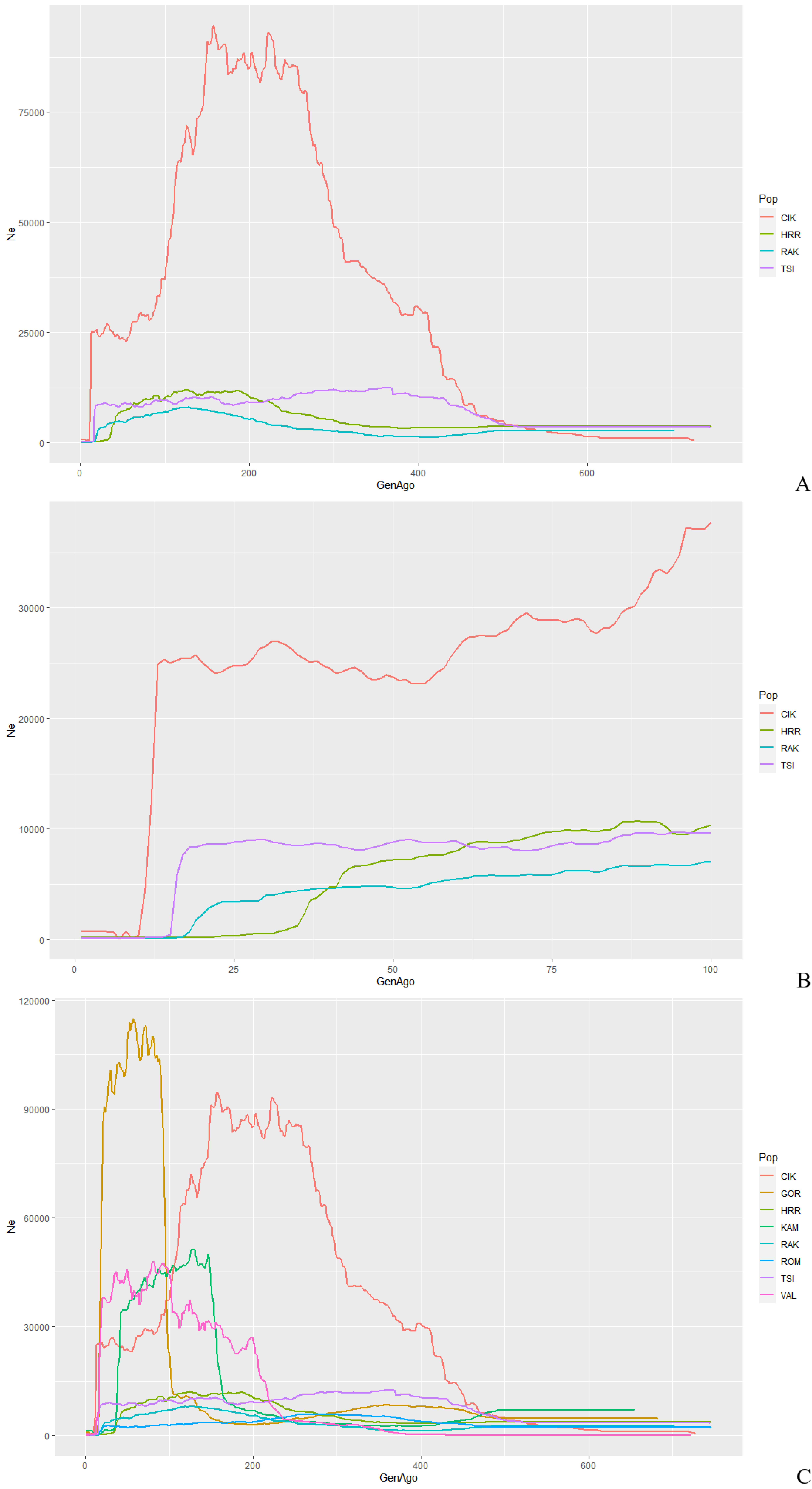

Supplement: Supplementary file 15 [file Image9.png]

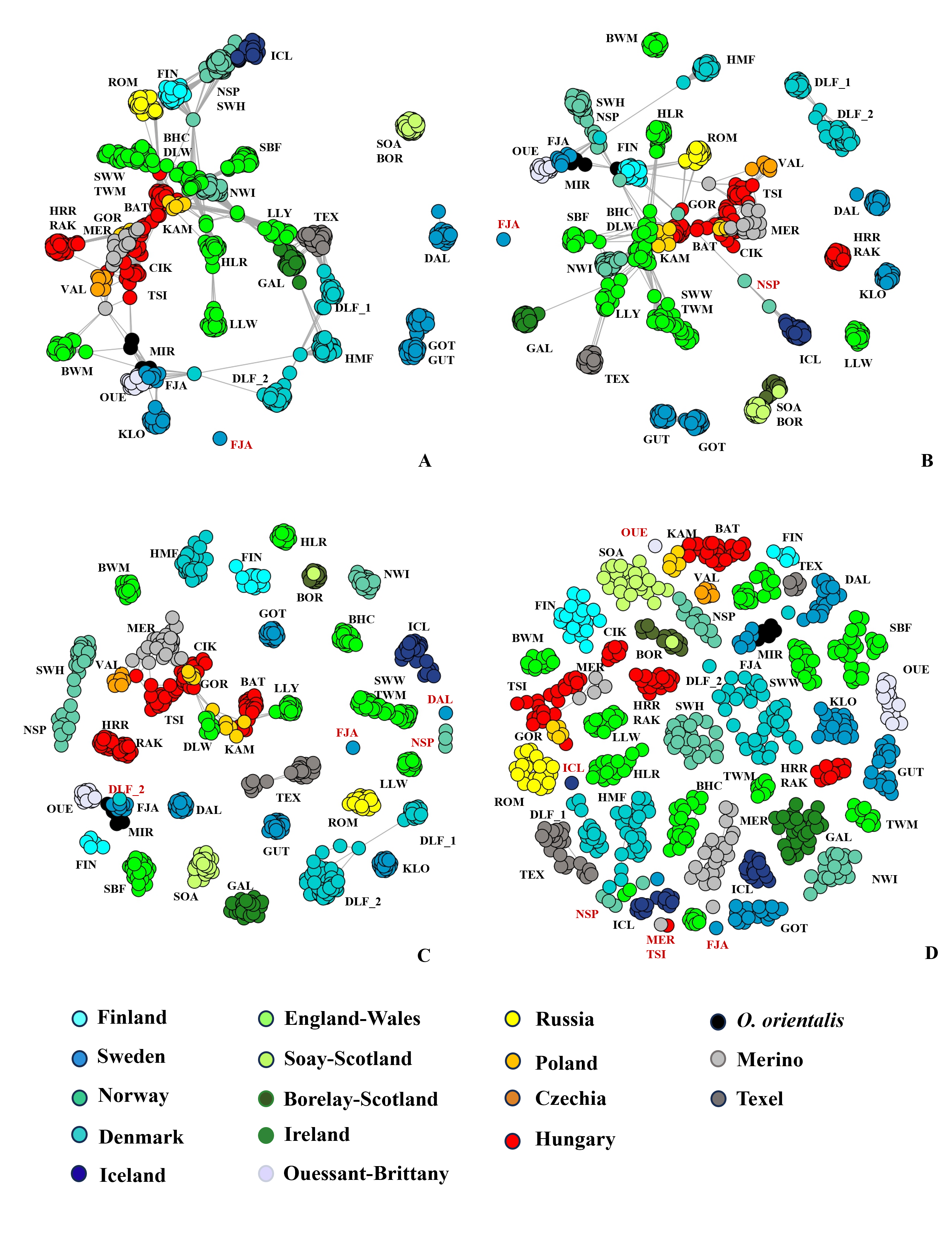

Supplement: Supplementary file 16 [file Image8.tif]

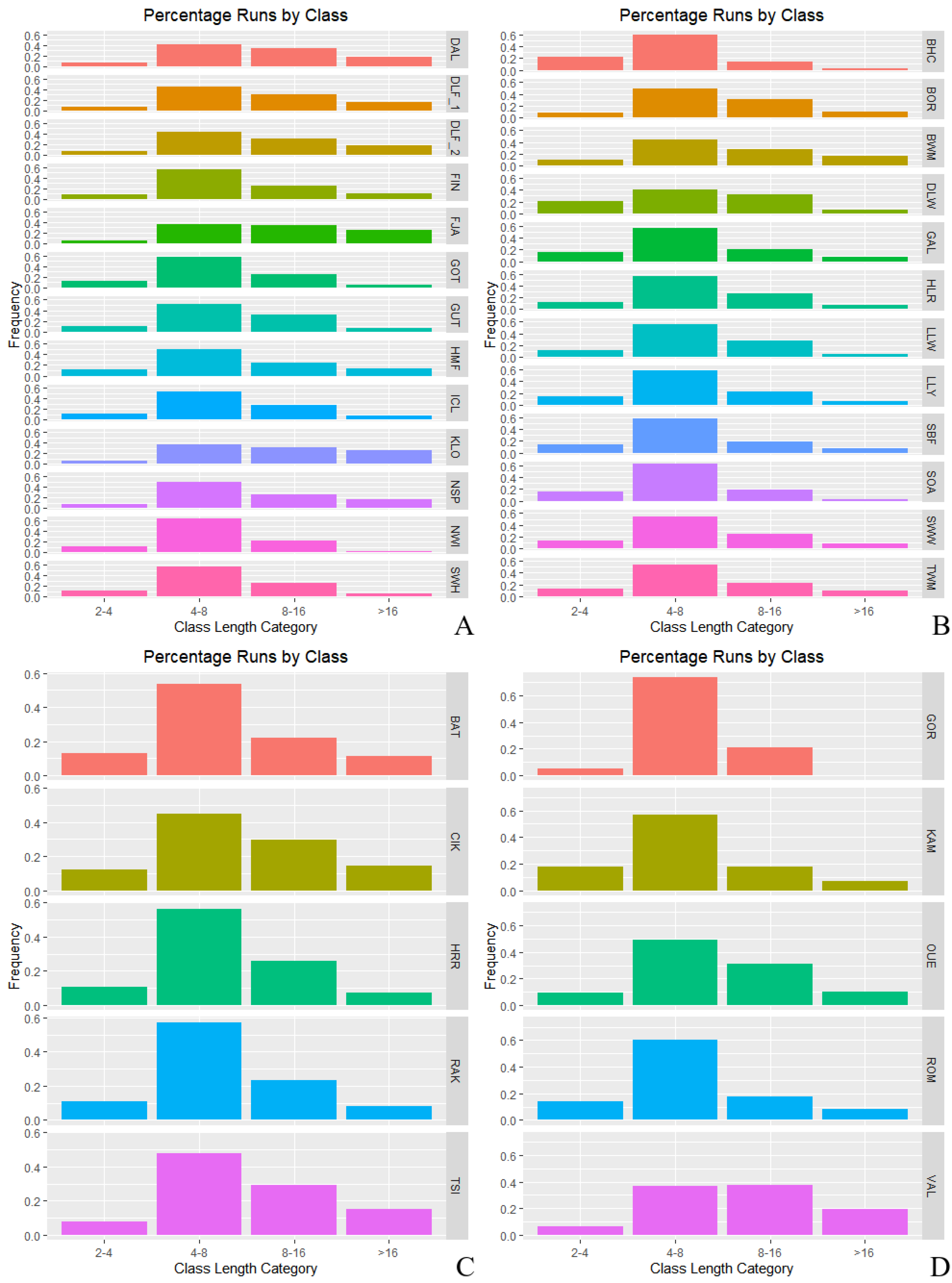

Supplement: Supplementary file 18 [file Image3.png]

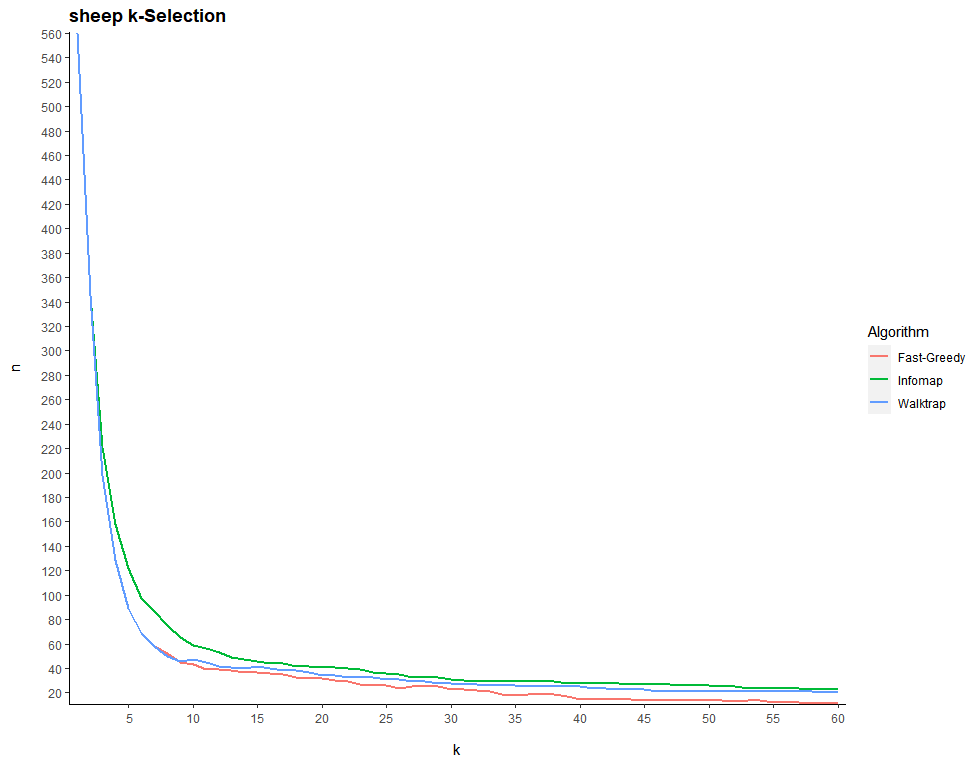

Supplement: Supplementary file 19 [file Image7.tiff]
