# Supplementary material for: Exploring the complex population structure and admixture of four local Hungarian sheep breeds
Source: Front Genet. 2025 Mar 19;16:1507315. doi: 10.3389/fgene.2025.1507315 (PMC11962792; doi:10.3389/fgene.2025.1507315)
Supplement: Supplementary file 6 [file Presentation1.pdf]

Supplementary Material

Image 1. Sampling map for the 36 target breeds. Breed code as in Supplementary Table 1.

Image 2. Pairwise F_{ST} estimates for each population represented by means of a heatmap.

Image 3 A-D. ROH analyses. Distribution of ROH per class of length. Four classes are plotted (2-4, 4-8, 8-16 and >16 Mb). From A to D: Scandinavian breeds, British Isles breeds, Hungarian breeds and the rest of Eastern European and Continental groups.

Image 4 A-D. Sum of ROH (SROH). Plot of the individual total coverage of ROH events for each population. From A to D: Scandinavian breeds, Irish-British Isles breeds, Hungarian breeds and the rest of eastern European breeds.

Image 5. Principal Component Analysis and the percentage of variance for the first two PCs, including all the breeds. Breed code as in Supplementary Table 1.

Image 6 A-C. Evaluation of model fit of inferred admixture proportions for the optimal K-value. A) Plot of cross-validation results to choose the optimal values of K; B) Admixture proportions inferred with ADMIXTURE assuming K=31 for all populations from this study; C) To evaluate the admixture model results performed with EvalAdmix by pairwise correlation of residuals matrix between individuals. The correlation will be close to 0 in case of a good fit of the data to the admixture model.

Image 7. Plot of the three machine learning methods (FastGreedy, Infomap and Walktrap) to establish the mutual nearest-neighbour (k) threshold in complex population studies and select the k according to the focus of the analysis.

Image 8 A-D. Network representing the genetic relationship among breeds as output of NetviewP. The figure illustrates the networks for four selected k, for a more complete overview of the complex population structure and to highlight the presence of substructure in our populations.

Image 9. Historical estimates of the effective population size obtained with the software GONE. A) plot for the Hungarian breeds and B) focus on the breeds from Eastern Europe. Breed code as in Supplementary Table 1.

Data sheet1 and Data sheet 2. Admixture graphs. A and B are the consensus trees at 75% and 95% of posterior probability, respectively.

Supplementary Table 1. Breed and dataset information.

Supplementary Table 2. Descriptive statistics. Observed, expected heterozygosity, and Genomic inbreeding coefficient (FROH), with the Confidence interval and quartiles indicated for each population/breed.

Supplementary Table 3. Pairwise F_{ST} and Reynolds' distance matrices.

Supplementary Table 4. Admixture proportions for the Hungarian breeds analysed in this study.

Supplementary Table 5. GCS scores (NetView analysis).

Supplementary Table 6. GONE Ne estimates considering the first 10 generations.
