# Supplementary material for: Exploring the complex population structure and admixture of four local Hungarian sheep breeds
Source: Front Genet. 2025 Mar 19;16:1507315. doi: 10.3389/fgene.2025.1507315 (PMC11962792; doi:10.3389/fgene.2025.1507315)
Supplement: Supplementary file 8 [file Table7.docx]

Supplementary Material

Supplementary Figure S1. Sampling map for the 36 target breeds. Breed code as in Supplementary Table 1.

Supplementary Figure S2. Pairwise F_ST_ estimates for each population represented by means of a heatmap.

Supplementary Figure S3 A-D. ROH analyses. Distribution of ROH per class of length. Four classes are plotted (2-4, 4-8, 8-16 and >16 Mb). From A to D: Scandinavian breeds, British Isles breeds, Hungarian breeds and the rest of Eastern European and Continental groups.

Supplementary Figure S4 A-D. Sum of ROH (SROH). Plot of the individual total coverage of ROH events for each population. From A to D: Scandinavian breeds, Irish-British Isles breeds, Hungarian breeds and the rest of eastern European breeds.

Supplementary Figure S5. Principal Component Analysis and the percentage of variance for the first two PCs, including all the breeds. Breed code as in Supplementary Table 1.

Supplementary Figure S6 A-C. Evaluation of model fit of inferred admixture proportions for the optimal K-value. A) Plot of cross-validation results to choose the optimal values of K; B) Admixture proportions inferred with ADMIXTURE assuming K=31 for all populations from this study; C) To evaluate the admixture model results performed with EvalAdmix by pairwise correlation of residuals matrix between individuals. The correlation will be close to 0 in case of a good fit of the data to the admixture model.

Supplementary Figure S7. Plot of the three machine learning methods (FastGreedy, Infomap and Walktrap) to establish the mutual nearest-neighbour (k) threshold in complex population studies and select the k according to the focus of the analysis.

Supplementary Figure S8 A-D. Network representing the genetic relationship among breeds as output of NetviewP. The figure illustrates the networks for four selected k, for a more complete overview of the complex population structure and to highlight the presence of substructure in our populations.

Supplementary Figure S9. Historical estimates of the effective population size obtained with the software GONE. A) plot for the Hungarian breeds and B) focus on the breeds from Eastern Europe. Breed code as in Supplementary Table 1.

Supplementary Figure S10. Admixture graphs. A, B and C) The consensus trees at 75%, 95% and 99% of posterior probability, respectively.
